# Multipolar Atom Types from Theory and Statistical Clustering (MATTS) Data Bank: Restructurization and Extension of UBDB

**DOI:** 10.1021/acs.jcim.2c00144

**Published:** 2022-08-09

**Authors:** Kunal Kumar Jha, Barbara Gruza, Aleksandra Sypko, Prashant Kumar, Michał Leszek Chodkiewicz, Paulina Maria Dominiak

**Affiliations:** Biological and Chemical Research Centre, Department of Chemistry, University of Warsaw, ul. Żwirki i Wigury 101, 02-089 Warszawa, Poland

## Abstract

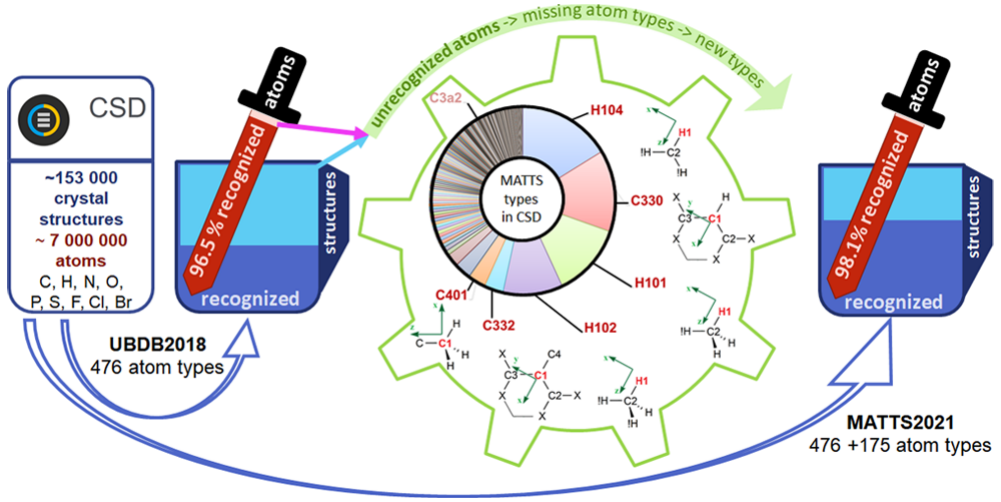

A fast and accurate operational model of electron density is crucial in many scientific disciplines including crystallography, molecular biology, pharmaceutical, and structural chemistry. In quantum crystallography, the aspherical refinement of crystal structures is becoming increasingly popular because of its accurate description in terms of physically meaningful properties. The transferable aspherical atom model (TAAM) is quick and precise, though it requires a robust algorithm for atom typing and coverage of the most popular atom types present in small organic molecules. Thus, the University at Buffalo Databank (UBDB) has been renamed to the Multipolar Atom Types from Theory and Statistical clustering (MATTS) data bank, broadened, restructured, and implemented into the software DiSCaMB with 651 atom types obtained from 2316 small-molecule crystal structures containing C, H, N, O, P, S, F, Cl, and Br atoms. MATTS2021 data bank now covers most of the small molecules, peptides, RNA, DNA, and some frequently occurring cations and anions in biological, pharmaceutical, and organic materials, including the majority of known crystal structures composed of the above elements. The multipole model parameters (*P*_val_, κ, κ′, *P*_*lm*_) obtained for different atom types were greatly influenced by neighboring atom types, hybridization, geometrical strain in the ring system, and charges on the molecule. Contrary to previous findings, the atoms showing variable oxidation states and ions deviate from the linear dependence of monopole-derived charges on the expansion–contraction κ parameter.

## 1 Introduction

For many decades, standard X-ray crystallography has been a very popular tool for structure determination but has been limited mostly to a spherically symmetric and isolated atomic electron density model (Independent Atom Model, IAM), from which we obtain information only about nuclei positions. In reality, molecules have electronic density (valence electron density) shared between bonded and interacting atoms. Atomic contributions to molecular electron densities are not spherical, and atoms in molecules are not neutral. This is not accounted for using spherical atom models.^1^ Electron densities of crystalline systems, by far more accurate than IAM, can be obtained in a quantitative manner by measuring ultra-high-resolution (*d* ≤ 0.5 Å) X-ray diffraction data.^1−3^ The high-resolution data thus obtained are modeled using Hansen and Coppens Multipole Model (pseudoatom) formalism (HCMM).^4^ In the HCMM, the molecular electron density is represented as the sum of pseudoatom densities (eq 1) composed of a spherical core and valence electron densities with an expansion of atom-centered real spherical harmonic functions:
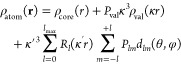
1

The first component accounts for the frozen core electron density ρ_core_. The second component accounts for the spherical expansion and contraction (determined by the parameter κ) of the valence shell ρ_val_ as well as the population of electrons in the valence shell (*P*_val_). The third component describes the aspherical deformation of the valence electron density through mathematical functions, in terms of real spherical harmonics (*d*_*lm*_) together with the radial terms (*R*_*l*_). The radial terms are modified by the expansion and contraction parameter κ′. The populations of spherical harmonics are identified by the parameters *P*_*lm*_. Pseudoatom functions are described in terms of polar coordinates (*r*, θ, and ϕ) defined on a local coordinate system centered on each atom. Further analysis of the multipole model, for example, by Bader’s approach of Quantum Theory of Atoms In Molecule (QTAIM), results in electron-density-based bonding properties.^5^ These results thus obtained are useful in understanding interaction-based phenomena in the solid state materials and macromolecules.^6,7^ However, growing a good-quality diffracting crystal and obtaining good-quality, high-resolution charge density data is a very time-consuming and tedious process. The multipolar modeling often is not reliable due to the poor quality of data compromised with experimental errors, model limitations, and phase problems.

A more runtime-efficient and simpler approach is the use of dummy bond electron or interatomic scatterers (IAS) along with the IAM, named the IAM-IAS model.^8^ Contrary to the multipole model, the IAM-IAS model does not replace the IAM. Instead, it treats the delocalized density as spherical Gaussian scatterers located at the centroid of the delocalized density and keeps the conventional spherical atoms unchanged.^9^ Thus, the information obtained from the IAM-IAS model is more detailed than IAM but simpler than the multipole model. The model was applied to both small-molecule and macromolecular systems.

The pseudoatom (multipole model) parameters (*P*_val_, κ, κ′, *P*_*lm*_) were found to be transferable; i.e., the parameters were found to be equivalent in similar chemical environments.^10^ It is possible to construct a multipole model for a new molecule with some previously known pseudoatom electron densities. The early works were based on the transfer of experimentally obtained pseudoatom parameters from one compound to construct the aspherical structural model of another compound, which was difficult to model using conventional experimental multipolar modeling.^10−12^ This research led to the advent of several pseudoatom data banks. Three such established data banks are the Experimental Library of Multipolar Atom Models (ELMAM/ELMAM2),^11,13^ the Invariom data bank,^14−17^ and the University at Buffalo Pseudoatom Data Bank (UBDB).^18−21^ With the availability of these data banks, it is possible to construct the transferable aspherical atom model (TAAM) and replace the conventional IAM in the crystal structure refinement. TAAM refinement is able to precisely and accurately determine atomic positions (especially for hydrogen atoms), describe electron density deformations due to lone pairs and bonding, and deconvolute thermal motion from static electron density more accurately than IAM.^16,22−25^

Each pseudoatom data bank is constructed slightly differently. The ELMAM/ELMAM2 data bank was derived from experimental charge density scattering factors obtained from high-resolution experimental data collected on small peptides and amino acids.^11,12^ The data bank was further extended to other small and macromolecules with the availability of more high-resolution experimental charge density data.^13^ The ELMAM/ELMAM2 data bank has found applications in deriving the aspherical model of small molecules, amino acids, peptides, and macromolecular proteins.^26−28^ The Invariom data bank and UBDB are based on the analysis of theoretical electron densities calculated for small and medium-sized molecules. The significant features of calculated static data are the absence of any systematic, random, or experimental errors and temperature and phase problems. In the Invariom data bank, the Invarioms (invariant atoms) are assigned to chemically unique atoms based on the nearest-neighbor approach having the same element and bond order.^14^ The next nearest neighbors are most often replaced by hydrogen atoms. The electron densities of Invarioms were generated from quantum chemical approximation while their transferability was found to be very accurate.^29^ The Invariom data bank has been generalized to most of the common elements such as C, H, N, O, S, F, Cl, P, and Si and has been utilized in various studies on small molecules, amino acids, and peptides.^15,29−31^ In the case of UBDB, the theoretical electron densities are calculated on the experimental geometry of a set of model molecules obtained from the Cambridge Structural Database (CSD)^32^ while chemically similar atoms are grouped into families with closely related pseudoatom populations and expansion–contraction parameters. UBDB is also based on the nearest-neighbor approach while in specific atom types the second and third neighbors are also defined. Unlike Invariom, the next nearest neighbors are kept as they are in the model molecule. The electron density reconstructed with the help of pseudoatom data banks is accurate enough not only to see benefits of replacing IAM by TAAM in structure refinements on modern diffraction data but also to model in a fast and quantitative manner, various electrostatic properties of molecules including biological macromolecular systems or a large set of small molecules. Electrostatic intermolecular interaction energies (Ee’s), for example, computed from UBDB-based electron densities are by far more accurate than the ones from point-charge approximations commonly used in molecular mechanics and approach the accuracy of quantum chemical calculations with much shorter computational time.^33^ This is because asphericity of charge distribution and charge penetration is taken into account.^34^ With Ee’s well balanced, much richer information on a drug molecule interactions with its protein partner can be obtained.^35,36^ Molecular electrostatic potentials reconstructed with UBDB are chemically accurate not only at the van der Waals region but also in the entire volume of a molecule.^21^ Therefore, the data bank may help in the near future to better model the experimental potentials measured with single-particle cryo-EM techniques.

Databanks based on interatomic scatterer modeling were also proposed,^37,38^ and their exemplary applications to crystal structure refinement and molecular electrostatic properties estimations were shown.

Our focus now was to extend the UBDB, restructure it, include a new software implementation, allow for the assignment of more diverse atom types with a more reliable algorithm, and hence improve the reach of modeling toward more diverse chemical structures and fields of application. The early developments of UBDB were mainly focused on modeling proteins, nucleic acids, peptides, and druglike molecules,^19−21,39^ and the software had limited capabilities to integrate with software from outside of the original quantum crystallography field. To overcome the limitations, we designed a new structure and a new file format for a pseudoatom data bank as a part of a new software library called DiSCaMB.^40^ The UBDB2018^21^ was translated to the new structure, and the data bank was renamed the Multipolar Atom Types from Theory and Statistical clustering (MATTS) data bank. The newly created MATTS data bank has already been used for determining the accurate and precise atomic positions of nonhydrogen and hydrogen atoms from standard X-ray diffraction experiments,^25^ and its usability in TAAM refinements of 3D electron diffraction (3D ED, microED) data was explored.^41^

In the presented work, the newly created MATTS data bank was widely extended to cover most of the atom types present in crystal structures deposited in the Cambridge Structural Database (CSD) and consisting of C, H, N, O, P, S, F, Cl, and Br atoms. The MATTS2021 data bank now allows a full model of electron densities of most known organic small molecules and determined crystal structures, including amino acids, peptides, nucleic acids, and proteins. Here, we present a detailed description of the new data bank structure, atom type algorithm, and the new entry parametrization. We describe how we optimized the procedure of defining the missing atom types to minimize the percentage of unrecognized atoms (atoms for which no atom type is present in the data bank). We also perform a basic analysis of the content of the new data bank by presenting various statistics based on selected multipole model parameters. A deeper analysis, with the use of statistical clustering methods, is presented in ref (61).

## 2 Methods

### 2.1 New Structure of the Data Bank: Atom Type Definitions

**Scheme 1:**
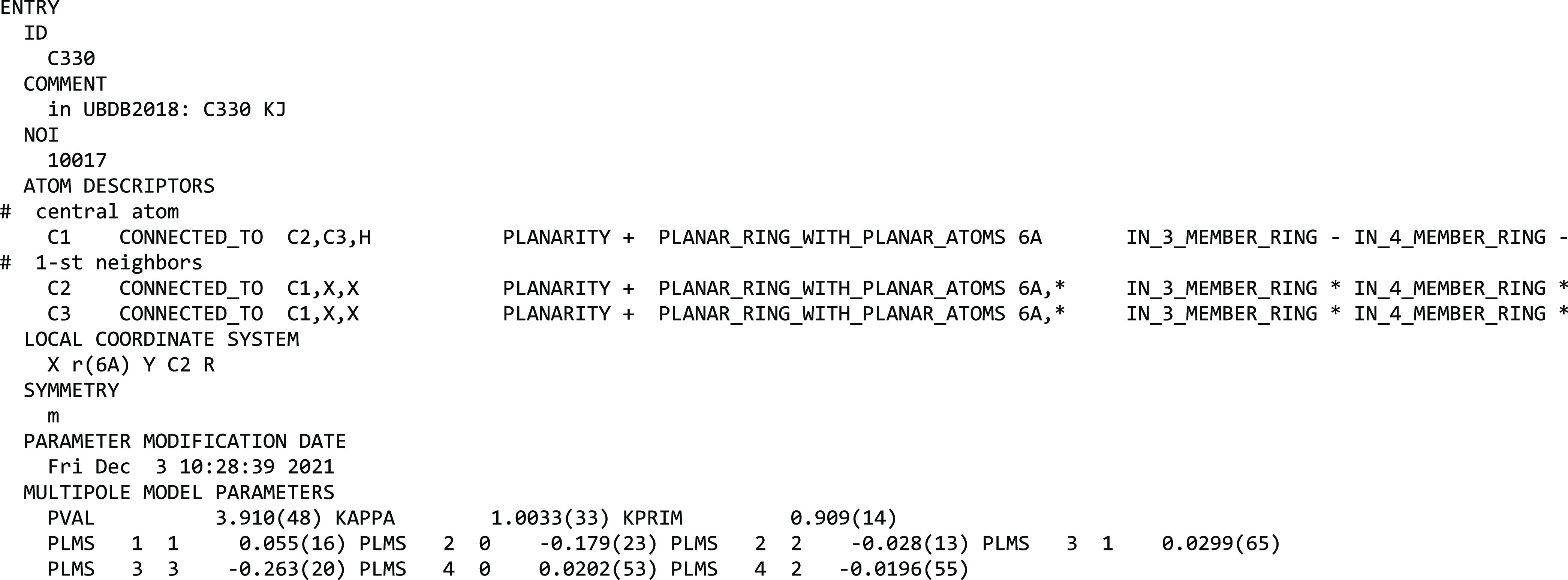
Format of the MATTS2021 Data Bank

The multipole model parameters (*P*_val_, κ, κ′, *P*_*lm*_), with mean values resulting from averaging over model atoms fulfilling the atom type definition, constitute the prime information stored in the pseudoatom data bank. The atom type definition in the MATTS2021 data bank is based on a set of connectivity rules that defines the environment of the central atom, enriched by information about element type, group planarity, ring size, and ring planarity. Unlike the UBDB and the LSDB software implementation associated with it, the connectivity list is now explicitly defined for each atom type and directly stored in the data bank. Information on the orientation of the local coordinate system with respect to neighboring atoms, along with the local symmetry associated with multipole functions, is stored in the data bank directly as well, in contrast to the previous implementation. The format of the MATTS2021 data bank is shown in Scheme 1. The details of each term in the data bank entry are given in the SI.

### 2.2 Local Coordinate System and Local Symmetry Assignment

Assignment of a local coordinate system to an atom type is an essential requirement of the pseudoatom data bank. The multipole model parameters of equivalent pseudoatoms should be represented in an analogous local frame to achieve consistency in expression of spherical harmonics functions centered at these atoms. Only then, the averaging of *P*_*lm*_ parameters will allow the extraction of maximum information about the electron density common to all averaged atoms. Orientation of the local coordinate system is also inevitably related with local symmetry. For a given symmetry, specific selection rules may apply, and characteristic multipoles may vanish (their *P*_*lm*_ values become zero), provided that the local coordinate system is oriented in a way that is optimal for that symmetry. The local (pseudo)symmetry of an atom type is predetermined by the character of its close neighbors and the character of chemical bonds. However, it is the local (pseudo)symmetry of an atom type electron density, not atom type topology, that is evaluated and then assigned to that atom type. Only with a proper orientation of local coordinate systems can the local (pseudo)symmetry of an atom type be revealed from the values of multipole model parameters.

Previously, at each stage of data bank creation and application, the local coordinate system was assigned by the program LSDB^42^ using a procedure that was independent from atom type definition. In fact, LSDB is a multipurpose program, includes a fully automatic independent procedure for the definition of unique local coordinate systems based on the coordination environment of each atom, and thus is an essential tool in experimental charge density investigations based on multipolar refinement with high-resolution diffraction data. Information about assigned local coordinate systems was not directly stored in the UBDB file. In most cases, this behaved properly, but in the case of more exotic atom types, the errors appeared to be related to the variable orientation of the axes with respect to neighbors while transferring the parameters from model molecules to the data bank for averaging, or while transferring them from the data bank to an investigated molecule at the data bank application stage.

Within the new MATTS2021 data bank format, the definition of local coordinate systems is a part of the atom type definition stored in the data bank. The local coordinate systems are defined manually while the new atom type entry is created. The local coordinate system defined in the atom type definition may differ from the local coordinate systems used during the refinement of individual atoms of model molecules. While atom type is parametrized from model molecules, the software from the DiSCaMB library recognizes how the local coordinate system was oriented in the model molecule and, if needed, reorients it to the one defined in the data bank entry. Reorientation of local coordinate systems is done before averaging multipole parameters. In this way, the creator of a new entry has an easy tool to check which coordinate system gives the highest transferability of multipole parameters (the lowest standard deviations of parameter values). Local symmetry can now also be set at the stage of atom type entry creation and might be independent from local symmetry constrains used during the refinement of model molecules. However, care must be taken to ensure that the local symmetry used during the refinement is of the same point group, or of lower symmetry (subgroup), as compared to the one set in the atom type definition. Further details about the local coordinate system assignment can be found in the SI.

Local symmetry is set by providing the symbol of one of the possible point groups (*m*, *mm*2, 3*m*, 4̅3*m*, etc.) or by writing “no” (meaning the 1 symmetry point group). Currently, the symmetry is assigned manually on the basis of similarities among topologies of the first neighbors, and care is taken in defining a new atom type so that it is consistent with values of multipole model parameters.

### 2.3 Atom Type Assignment Algorithm

The atom type definition corresponds to a molecular graph, with atom vertices “colored” with atomic descriptors. An atom type assignment algorithm, implemented in the DiSCaMB library and strictly associated with the MATTS2021 data bank, utilizes the graph representation of an atom type. An atom type is assigned by performing a graph–subgraph isomorphism check using the VF2 subgraph isomorphism algorithm^43^ as implemented in the vflib graph matching library, v 2.0.

The main idea behind the atom type assignment (matching, perception) algorithm can be summarized with the following points:

1. Each atom type *t* is represented as a graph *G*_*t*_ with vertices corresponding to atoms, edges corresponding to bonds, and atomic descriptors (such as atomic number, planarity, etc.) corresponding to components of “colors” of the vertices.
2. An algorithm for atom type assignment works on a molecule (i.e., finite set of atoms) hereinafter referred to as M. Therefore, it is necessary to extract a subset of atoms from a crystal structure for further processing. This set of atoms consists of atoms of an asymmetric unit and their neighbors up to *n*th neighbor, where *n* is the smallest number which is sufficient for correct assignment of atom types. A connectivity of atoms in the crystal is represented as a periodic graph. For each atom in an asymmetric unit, a set of its neighbors up to the *n*th one is calculated with a breadth-first search algorithm, and a union of the sets forms the molecule M.
3. Similarly as for atom types, atoms and bonds of M are represented with graph *G*_*M*_.
4. For each atom *a* of M that belongs to an asymmetric unit, a subgraph *G*_*a*_ of *G*_*M*_ consisting of the atom *a* and its neighbors up to *n*th one is formed.
5. If atom *a* is of type *t*, then *G*_*t*_ must be a subgraph of *G*_*a*_. This is tested by searching for graph–subgraph isomorphism using the VF2 algorithm. If a match is found, i.e., a mapping from vertices of *G*_*t*_ to vertices of *G*_*a*_ is found, then an additional check has to be performed. Atom type specifications allow a definition of whether some atoms in the atom type definition belong to the same or different rings and also verification if the match fulfils this aspect of the atom type definition or requires an additional test.
6. For each atom of M that belongs to an asymmetric unit, an attempt to match each atom type in the data bank is undertaken until the match is found or until all of the atom types are examined.
7. Essentially, some atom types can be a more general version of other atom types. In such a situation, the more general atom type should be specified in the data bank file after the more detailed one, since then, the more detailed one will be tested first.

### 2.4 Data Bank Construction

#### 2.4.1 Search for the Most Popular Missing Atom Types

To find the most popular atom types that frequently exist in small-molecule crystals but are still missing in the MATTS data bank, we focused our attention on the largest available database of organic and organometallic crystal structures, the Cambridge Structural Database (CSD). We retrieved from the CSD a subset of structures containing only those chemical elements for which we aimed to have all possible atom types in the MATTS2021 data bank: C, H, N, O, P, S, F, Cl, and Br. We processed the entire subset in order to identify atoms to which none of the already existing atom types was assigned. We categorized unassigned atoms into groups of atoms having similar topological features and sorted them according to the size of the group, thus identifying the most popular groups. Groups with the largest number of unassigned atoms were starting points for introducing new atom types to the MATTS2021 data bank. The details of entire procedure were as follows.

The CSD (Nov. 2019) search was performed with the ConQuest program^44^ and was limited to structures which contained a desired set of chemical elements. All possible combinations of C and one or more of H, N, O, P, S, F, Cl, and Br elements were considered. As search criteria, queries determining the following features were used: (a) a list of elements that must be present in same structure, (b) a list of excluded elements, and (c) the C–H distance to ensure the presence of coordinates for at least one hydrogen atom for structures allowed to have hydrogen. The filters available in ConQuest such as 3D coordinates determined, *R*-factor lower than 5%, nondisordered structures, no unresolved errors, only single crystal structures, and only organics were applied to avoid the selection of crystal structures with the wrong geometry or missing atoms. The resulting subset still contained structures with missing or disordered hydrogen atoms. Custom-made filters were applied to remove most of the structures with problematic hydrogen atoms.

The selected subset of the CSD was next analyzed by a new utility program, typeQuest, created from the developer version of the DiSCaMB software library.^40^ typeQuest read atom coordinates from .cif or SHELX.res format files, characterized topological features of each atom, and, if possible, assigned atom types from the provided MATTS2021 data bank file to the analyzed atoms. Unassigned atoms were further arranged by the program into groups of atoms having similar topology, i.e., having the same atomic number, the same type and number of chemical elements among first neighbors, the same type and number of chemical elements among second neighbors, and the same values of the remaining central atom descriptors such as group planarity or belongingness to various rings. In addition, typeQuest provided appropriate statistics documenting (a) how many atoms were assigned to which atom type, (b) the total number and fraction of all assigned atoms in relation to all analyzed atoms, (c) the total number and fraction of structures with all assigned atoms in relation to all analyzed structures, and (d) how many unassigned atoms belong to each identified group. The statistics along with detailed information about assigned and unassigned atoms were printed to various log files.

On the basis of topological features of highly populated groups of unassigned atoms, new entries to the MATTS2021 data bank were manually defined. While setting the definitions, decisions about the level of details of the first and the second neighbors to be included in the definition had to be arbitrarily made. Decisions were made on the basis of knowledge gained while building UBDB. The number and element type of the first neighbors were preserved. The total number of second neighbors was usually preserved as well, but element types of second neighbors were often ignored. Next, electron density pseudoatom parameters for each atom type entry were computed following the procedures given in section 2.4.2 on the basis of 15–20 manually selected model molecules. Care was taken to ensure that, among model molecules, the most frequently occurring combinations of chemical elements among the second neighbors were present. After careful analyses of the obtained multipole model parameters, the atom type definition was accepted or changed if, (a) for one (or more) multipole model parameter, a large sample standard deviation was observed, or (b) assigned local symmetry was not fulfilled by multipole model parameters. Thus, each arbitrarily set atom type definition was verified if it allows the attainment of an acceptable level of multipole model parameter transferability. The procedure was iteratively repeated from the very beginning until (a) all atom type definitions were accepted, and (b) the number of unassigned atoms in the most highly populated groups dropped significantly.

#### 2.4.2 Multipole Model Parameter Calculations on Selected Model Molecules

The MATTS2021 data bank was constructed following the procedure previously established for the older versions of UBDB.^19−21^ A set of good-quality crystal structures with molecules representing the missing atom types was retrieved from the CSD.^32^ The hydrogen bond distances (X–H, X being any nonhydrogen atom) in model molecules were extended to neutron bond distances using the LSDB program.^42^ Single-point calculations were performed on selected molecules using the GAUSSIAN03 program^45^ to obtain molecular wave functions. Density functional theory (DFT) with the standard split-valence double-exponential 6-31G** basis set with polarization functions^46^ and the B3LYP functional^47,48^ was used for the calculations. The theoretical valence-only structure factors were calculated in the range 0 < sin *θ/λ* < 1.1 Å^–1^ for reciprocal-lattice points corresponding to a cubic cell with 30 Å edges and the *P*1 space group. Subsequently, the obtained structure factors were fitted with the Hansen–Coppens multipole model^4^ using the method of least-squares in the XD program suite.^49^ Structure factor phases were constrained to the one obtained from the structure factors calculations. For the deformation functions of multipoles, default values of Slater radial function coefficients *n*_*l*_ were used except for sulfur and phosphorus atoms. *n*_*l*_ = (2, 4, 6, 8) and *n*_*l*_ = (6, 6, 6, 6) were applied for sulfur and phosphorus atoms, respectively, following the previously established procedure.^50^ The multipole expansion was truncated at the hexadecapolar level (*l*_max_ = 4) for the nonhydrogen atoms and at the quadrupolar level (*l*_max_ = 2) for hydrogen atoms, for which only bond-directed functions of *l*,*m* = 1,0 and 2,0 were refined. Both radial screening factors (κ and κ′) were refined independently for each atom, with the exception of the chemically equivalent hydrogen atoms that shared the same κ and κ′ parameters. Local symmetry constrains, set by the LSDB program, were applied except for atoms having 6̅2*m* (*D*_3*h*_), 4̅3*m* (*T*_*d*_), and *m*3*m* (*O*_*h*_) local symmetry. Atoms with 6̅2*m* symmetry were refined with the 3*m* constraints, and atoms with the 4̅3*m* and *m*3*m* symmetries were refined with no constraints.

The values of multipole model parameters thus obtained (*P*_val_, κ, κ′, *P*_*lm*_) were averaged over a family of chemically equivalent atoms using the bankMaker utility program from the DiSCaMB library and atom type definitions provided with the external data bank file. Whenever the local coordinate system of an atom used during the refinement was different from the one defined in the atom type definition, before averaging, the program rotated the multipole functions^51,52^ to the coordinate system defined for the atom type. Mean values of multipole model parameters and their sample standard deviations were computed for all of the parameters and were printed to the data bank file following the selection rules given in the SI.

Finally, definitions of all atom types, including those translated from the UBDB2018, were reexamined with the focus on large sample standard deviations for any of the multipole model parameters obtained from averaging over the whole set of model molecules. Care was taken so that, for most of the multipole model parameters, their sample standard deviations did not exceed their desired values: 0.1 e for *P*_val_ and κ′, 0.01 for κ, and 0.05 e for *P*_*lm*_. Some atom types were allowed to have larger sample standard deviations, if they represented atoms with expected large variations in electron density (e.g., delocalized bonds to the first neighbors), or atom types that were extremely rare in the analyzed subset of the CSD.

## 3 Results and Discussions

### 3.1 Atom Types in the Data Bank

The old atom types from UBDB2018^21^ were translated to the new format, modified to benefit from the new design, split if necessary, and reparametrized by reaveraging over atoms collected from the newly added model molecules along with the previous molecules. This resulted in 476 atom types imported to the MATTS2021 data bank from the UBDB2018. 175 new atom types were added following the specific needs of users and the here-described procedure of identifying the most popular missing types among crystal structures deposited in the CSD and containing exclusively one or more elements like C, H, N, O, P, S, F, Cl, and Br.

The MATTS2021 version of the extended data bank now contains 651 atom types, among which there are 29 hydrogen, 378 carbon, 104 nitrogen, 82 oxygen, 35 sulfur, 12 phosphorus, 6 chlorine, 3 fluorine, and 2 bromine atom types. A total of 2516 model molecules were used, and these were taken from 2316 crystals structures. The CSD refcodes of all of the structures are given in the SI. Each atom type resulted from averaging multipole model parameters of many individual atoms. We targeted to have ca. five representative atoms per atom type. Some atom types were very popular among model molecules and thus were parametrized on the basis of a much larger number of individual atoms. More than 50% of the types resulted from averaging 15 individual atoms or more. One third of the types are based on more than 30 atoms, whereas the record holders are the H104, H101, and C330 types, which are built from more than 10 000 atoms each. The achieved transferability errors, measured by sample standard deviations of multipole model parameters, were relatively low. More than 90% of the *P*_val_ parameters have their ssds in the range 0.01–0.1 e. For 90% of the κ parameters, ssd is in the range 0.001–0.01. In the case of κ′ parameters, more than 80% have their ssds in the range 0.01–0.1. Finally, only 11% of atom types have at least one *P*_*lm*_ with its ssd exceeding 0.05 e; usually, ssds are only slightly above the limit.

### 3.2 Subcategories of Atom Types in the Data Bank

Individual details of the atom types present in MATTS2021 are given below. The analysis is focused on hybridization type and on values of *P*_val_, κ, and κ′ parameters. A global analysis of *P*_*lm*_ values was not possible here and will be given in ref (61), as populations of multipoles are highly dependent on the chosen local coordinate system. In the current version of the MATTS2021 data bank, orientation of coordinate system in relation to the first neighbors varies from one atom type to another depending on the local symmetry assigned to the type.

#### 3.2.1 Hydrogen

**Figure 1:**
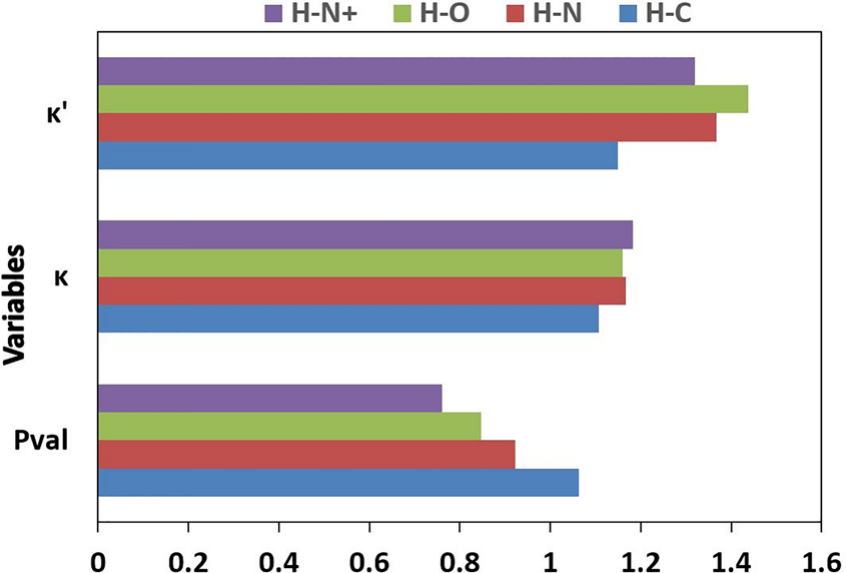
Mean values of *P*_val_, κ, and κ′ parameters for selected hydrogen types. H–C, H–O, H–N, and H–N^+^ indicate mean values of a given parameter obtained by averaging over all atom types of hydrogen atoms attached to carbon, oxygen, nitrogen, or four coordinated nitrogen (N^+^) atoms, respectively.

Hydrogen, being elusive to X-ray diffraction, is very difficult to model using the IAM refinement, and the description of a hydrogen atom position from IAM was found to be inaccurate. However, hydrogen atom location can be determined accurately and precisely on a wide range of structures using Hirshfeld atom refinement (HAR)^53^ and coupling HAR with the data bank of extremely localized molecular orbitals (ELMO-DB).^54,55^ It was also reported that aspherical TAAM refinement increased the bond lengths of hydrogen atoms toward more accurate values. However, it is very necessary to have all of the proper atom types with their neighboring atom types well-defined to achieve an accuracy approaching the reference neutron bond distances using TAAM. The MATTS2021 data bank contains 29 hydrogen atom types with a hydrogen atom attached to aliphatic and aromatic carbons and nitrogen, aliphatic and aromatic alcohol, carboxylic acids, esters, etc. The MATTS2021 data bank also contains hydrogen atom types attached to sulfur and phosphorus. Additionally, hydrogen atom types attached to hydronium and ammonium ions and fused water molecules were added to the MATTS2021 data bank. Thus, the TAAM refinement using the current version of the MATTS2021 data bank resulted in a precise and accurate determination of hydrogen atom positions, approaching reference neutron bond lengths with an accuracy similar to that of HAR.^25^ The valence populations (*P*_val_) for hydrogen types vary in the range from 0.58(1) e (H120, for hydrogen atoms in hydronium ions) to 1.10(9) e (H130, for hydrogen atoms in ethyne) and 1.09(4) e (H101, for hydrogen atoms in the methyl group). In general, the values of *P*_val_ and expansion and contraction κ and κ′ parameters for hydrogen atom types depend on the type of neighboring atoms attached to the hydrogen atom (Figure 1). The *P*_val_ parameters decrease in the order H–C > H–N > H–O > H–N^+^; κ parameters follow the order H–C < H–O < H–N < H–N^+^, and κ′ parameters change in the order H–C < H–N^+^ < H–N < H–O.

#### 3.2.2 Carbon

**Figure 2:**
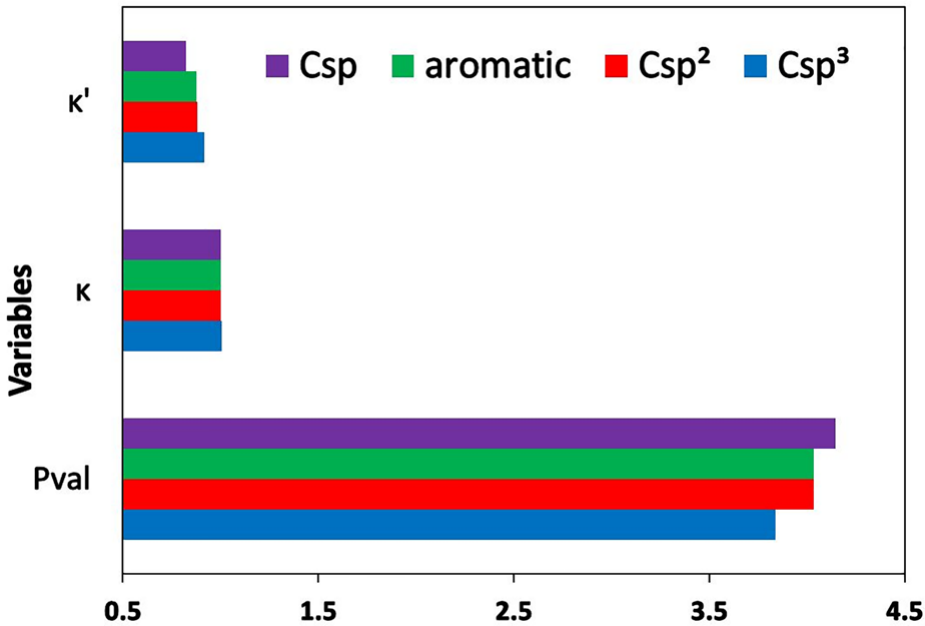
Mean values of *P*_val_, κ, and κ′ parameters across the carbon atom types depending on hybridization. C sp, aromatic, C sp^2^, and C sp^3^ indicate mean values of a given parameter obtained by averaging over all atom types of carbon atoms with the sp hybridization, sp^2^ hybridization being a part of an aromatic system, sp^2^ hybridization being part of an aliphatic group, and sp^3^ hybridization, respectively.

There are 378 atom types of carbon reported in the MATTS2021 data bank. 229 of them are for aliphatic carbon atoms, out of which 132 are for sp^3^ hybridized, 93 for sp^2^ hybridized, and 4 for sp hybridized carbon atoms. There are 149 atom types for aromatic carbon atoms, out of which 16 are on the border of two or three fused rings of various sizes (5-, 6-, or 7-membered rings). *P*_val_ for carbon atom types was found to be in the range of 4.47(5) e (maximum for C201, nitrile group) and 3.50(8) e (minimum for C410, methyl group attached to nitrogen). The expansion and contraction κ and κ′ parameters with maximum values were found for C410 [1.027(5)] and C783 [1.06(2), sp^2^ carbon atom attached to one sp^3^ carbon atom and two sulfur atoms from the (trifluoromethyl)sulfonyl groups], respectively. Minimum κ and κ′ parameter values were found for C314c [0.989(2), sp^2^ carbon atom attached to two sp^2^ carbon atoms and one hydroxyl group] and C201 [0.79(2)], respectively. Depending upon the hybridization, the values of the *P*_val_ parameters decrease in the order C sp > C sp^2^ > C sp^3^, while the values of κ parameters do not change significantly (Figure 2). The values of κ′ parameters increase in the order C sp < C sp^2^ < C sp^3^. The atom types for aromatic carbon atoms having the sp^2^ hybridization have similar values of parameters as atom types for the sp^2^ hybridized aliphatic carbon atoms.

**Figure 3:**
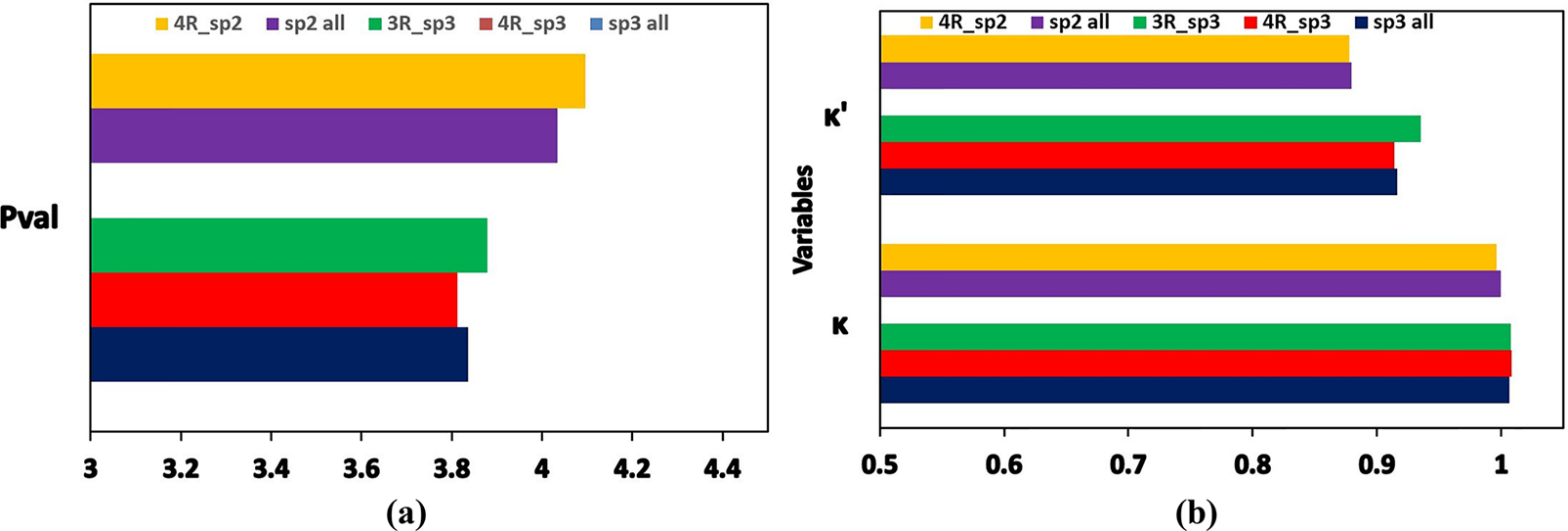
Mean values of (a) *P*_val_ and (b) κ and κ′ parameters across carbon atom types depending on hybridization and belonging to 3- or 4-membered rings. The mean values of a given parameter were obtained by averaging over: all atom types of carbon atoms belonging to 3-membered rings and having the sp^3^ hybridization (3R_sp3), belonging to 4-membered rings and having the sp^3^ hybridization (4R_sp3) or the sp^2^ hybridization (4R_sp2), all C sp^3^ atom types in the data bank (sp3 all), and all C sp^2^ atom types in the data bank (sp2 all).

The 3- and 4-membered ring carbon atom types represent highly strained systems. There are a total of 9 atom types for carbon atoms belonging to a 3-membered ring, and all of them are for the sp^3^ hybridized carbon atoms. The MATTS2021 data bank does not yet contain any carbon atom types for the sp^2^ hybridized carbon atoms belonging to the 3-membered rings. There are 22 atom types for carbon atoms belonging to 4-membered rings; out of those, 17 are for the C sp^3^ atoms, and 5 are for the C sp^2^ atoms. The 4-membered ring C sp^3^ atom types have lower values of *P*_val_ (Figure 3), whereas the 3-membered ring C sp^3^ types have higher values of *P*_val_ compared to the global mean value computed for all C sp^3^ atom types in the data bank. While there was no change observed for κ parameters, the κ′ parameters were found to have higher values for 3-membered ring C sp^3^ atom types than the global mean κ′ value (Figure 3). The values of *P*_val_ for atom types of sp^2^ hybridized carbon atoms in 4-membered rings were found to be higher than the global mean value for all C sp^2^ atom types, whereas the values for κ and κ′ parameters do not differ (Figure 3).

#### 3.2.3 Nitrogen

**Figure 4:**
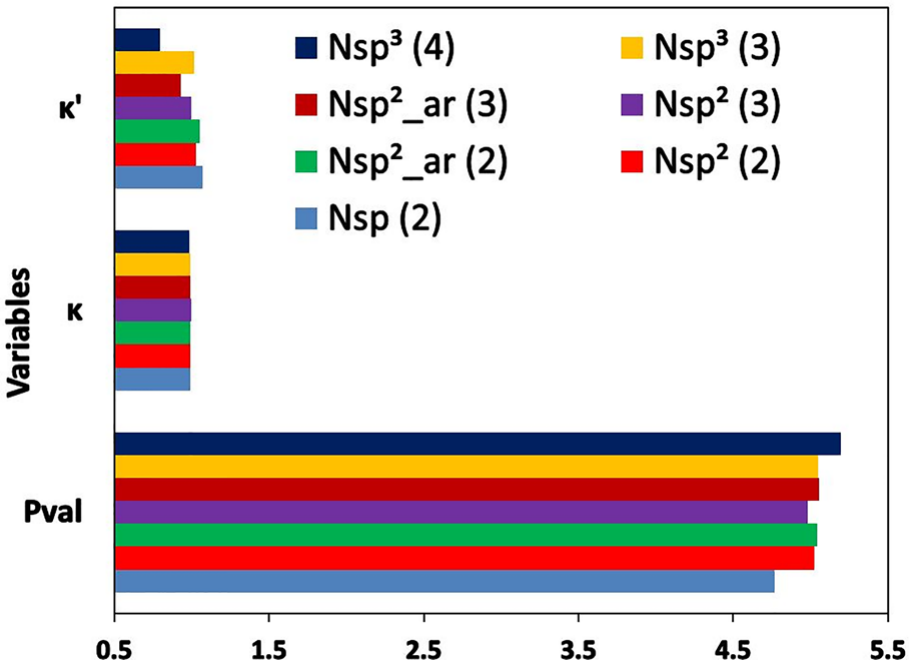
Mean values of *P*_val_, κ, and κ′ parameters across different atom types of nitrogen atoms categorized in seven different systems, namely, (1) N sp^3^ (4), for sp^3^ hybridized nitrogen atoms attached to four neighbors; (2) N sp^3^ (3), for sp^3^ hybridized nitrogen atoms attached to three neighbors; (3) N sp^2^_ar (3), for sp^2^ hybridized nitrogen atoms belonging to aromatic rings and attached to three neighbors; (4) N sp^2^ (3), for sp^2^ hybridized nitrogen atoms attached to three neighbors; (5) N sp^2^_ar (2), for sp^2^ hybridized nitrogen atoms belonging to aromatic rings and attached to two neighbors; (6) N sp^2^ (2), for sp^2^ hybridized nitrogen atoms attached to two neighbors; and (7) N sp (2), for sp hybridized nitrogen atoms attached to two neighbors.

The nitrogen atom types can be divided into seven categories, based on hybridization, number of neighbors, and belongingness to aromatic systems. The variation in values of *P*_val_, κ, and κ′ parameters for different categories of nitrogen atom types is shown in Figure 4. As found in other atom types, the values of κ parameters do not change across different categories of atom types. Visible differences were observed for *P*_val_ and κ′ parameters. The atom types for the sp hybridized nitrogen atoms connected to two neighbors have the lowest mean value of *P*_val_ (4.776 e) and the highest mean value of κ′ (1.073). The atom types describing the sp^3^ hybridized nitrogen atoms with four neighbors have the highest mean value of *P*_val_ (5.195 e) and lowest mean value of κ′ (0.796). The mean values of *P*_val_ and κ′ parameters for atom types of the sp^3^ hybridized aromatic nitrogen atoms with three neighbors are higher and lower, respectively, compared to atom types of analogous nitrogen atoms but from aliphatic groups. In the case of atom types describing sp^2^ hybridized nitrogen atoms with two neighbors, belongingness to aromatic or aliphatic groups does not influence the mean values of *P*_val_ and κ′.

#### 3.2.4 Oxygen

**Figure 5:**
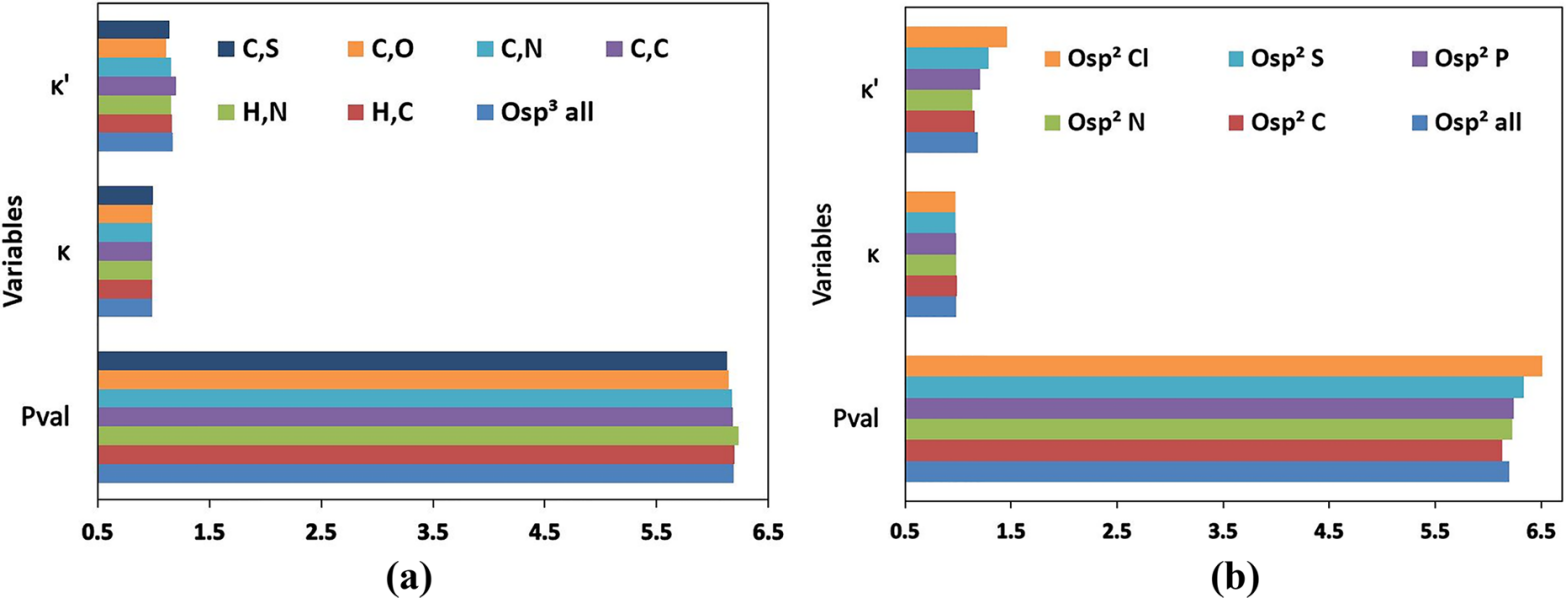
Mean values of *P*_val_, κ, and κ′ parameters across the atom types for oxygen atoms with sp^2^ or sp^3^ hybridization and attached to different atoms. (a) sp^3^ hybridized oxygen atoms attached to two neighboring atoms with more than one entry in the data bank [C—O—S (C,S), C—O—O (C,O), C—O—N (C,N), C—O—C (C,C), H—O—N (H,N), H—O—C (H,C), and all together (O sp^3^ all)]; (b) sp^2^ hybridized oxygen atoms attached to one neighboring atom [O=Cl (O sp^2^ Cl), O=S (O sp^2^ S), O=P (O sp^2^ P), O=N (O sp^2^ N), O=C (O sp^2^ C), and all together (O sp^2^ all)].

The oxygen atom types were divided into two categories, sp^2^ hybridized oxygen atoms attached to one neighbor (O sp^2^) and sp^3^ hybridized oxygen atoms attached to two neighbors (O sp^3^). There was no significant difference found in the mean values of *P*_val_, κ, and κ′ parameters computed for all O sp^2^ atom types when compared to all O sp^3^ atom types. A further analysis on the type of neighbors attached to O sp^3^ atom types reveals that there is no significant difference in the mean values of parameters (Figure 5a). However, in the case of O sp^2^ atom types divided into subcategories depending upon the type of the first neighbor, the parameters changed significantly. The *P*_val_ values decrease in the order O sp^2^ Cl > O sp^2^ S > O sp^2^ P > O sp^2^ N > O sp^2^ C (Figure 5b). The highest *P*_val_ and κ′ were found for the O sp^2^ Cl which represents oxygen atoms attached to chlorine atoms in perchlorate ions.

#### 3.2.5 Sulfur

**Figure 6:**
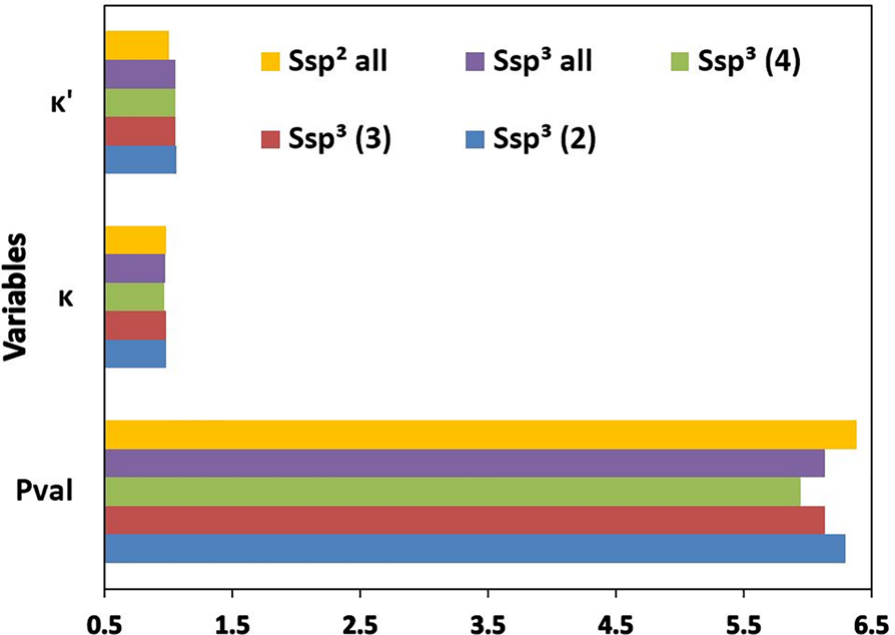
Mean values of *P*_val_, κ, and κ′ parameters across the atom types for sulfur atoms with sp^2^ or sp^3^ hybridization and attached to different numbers of neighboring atoms: sp^2^ sulfur atoms with one neighbor (S sp^2^ all); sp^3^ sulfur atoms with two [S sp^3^ (2)], three [S sp^3^ (3)], or four [S sp^3^ (4)] neighbors; and all together (S sp^3^ all). The mean values were computed only for categories of atom types for which there is more than one entry in the data bank.

The MATTS2021 data bank contains sulfur atom types for sp^2^ and sp^3^ hybridized sulfur atoms. The mean values of *P*_val_ and κ parameters for sp^2^ hybridized sulfur atom types (S sp^2^ all) are higher than for sp^3^ hybridized sulfur atom types (S sp^3^ all), while for κ′ parameters, the reverse is true (Figure 6). All of the sp^2^ hybridized sulfur atoms are connected to a single neighbor while sp^3^ hybridized sulfur atoms are attached to 2, 3, or 4 neighbors. Within the sp^3^ hybridized sulfur atom types, the mean values of *P*_val_ parameters vary depending upon the number of first neighbor atoms. The *P*_val_ parameters follow the order S sp^3^ (2) > S sp^3^ (3) > S sp^3^ (4). There is no significant difference between the mean values of κ and κ′ parameters across various S sp^3^ sulfur atom types.

#### 3.2.6 Phosphorus

Out of the 12 phosphorus atom types added to the MATTS2021 data bank, 11 of them have similar *P*_val_, κ, and κ′ values; the *P*_val_ values vary from 5.20(6) e to 5.42(2) e, κ parameters from that 0.932(3) to 0.948(3), and κ′ parameters from 1.021(8) to 1.094(4). In the case of PF_6_^–^, the phosphorus atom type (P601) has the lowest *P*_val_ (4.76(6) e) and κ′ (0.962(6)) values, whereas the κ (0.940(1)) parameter has a value similar to other those for phosphorus atom types.

#### 3.2.7 Halogen Atoms

**Figure 7:**
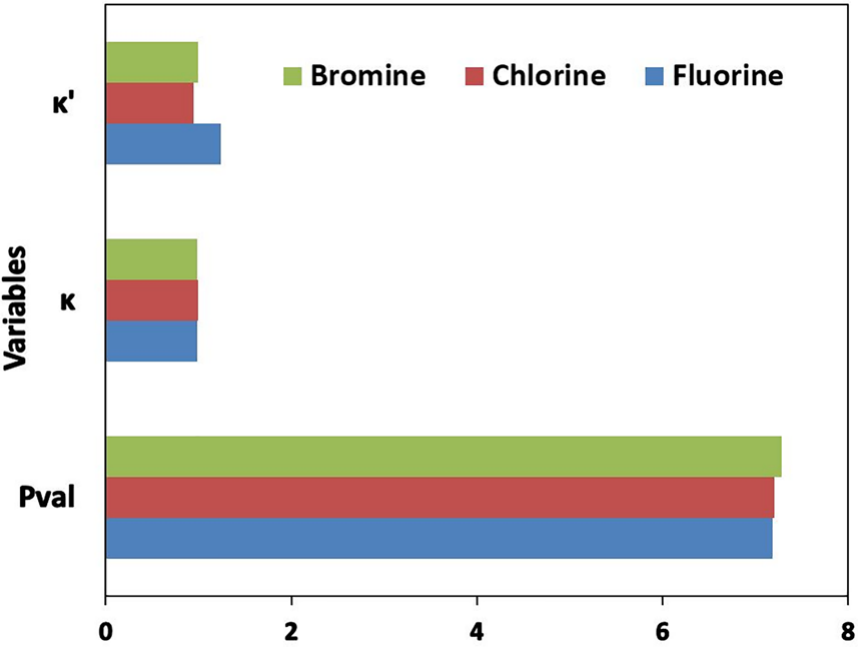
Mean values of *P*_val_, κ, and κ′ parameters across the atom types for halogen atoms: fluorine, chlorine, and bromine atom types having more than one entry in the data bank.

Halogen types include three fluorine atom types, six chlorine atom types, and two bromine atom types. The *P*_val_ values for all of the halogen atom types are consistent [range between 7.17(3) e and 7.3(1) e] except the ClO_4_ atom type for the ClO_4_^–^ ion which has the lowest *P*_val_ [6.127(3) e]. The κ parameters for all of the halogen atom types were found to be consistent and to vary from 0.985(2) to 0.9946(2). The κ′ parameters for fluorine atom types have the highest values, followed by those for bromine atom types, and the lowest values are observed for chlorine atom types. The κ′ parameter for the ClO_4_ atom type is the lowest [0.878(2)]. The mean values of *P*_val_ parameters for halogen types increase in the order F < Cl < Br; the mean values of κ parameters remain unchanged, while the mean values of κ′ parameters follow the order Cl < Br < F (Figure 7). Iodine could not be added to the MATTS2021 data bank at present because the DiSCaMB library uses atomic scattering factors based on the atomic wave functions of Clementi and Roetti,^56^ which has tabulated wave function of atoms up to krypton (Kr) and does not include iodine.

### 3.3 Variation in Monopole-Derived Charges and Expansion–Contraction Parameters

**Figure 8:**
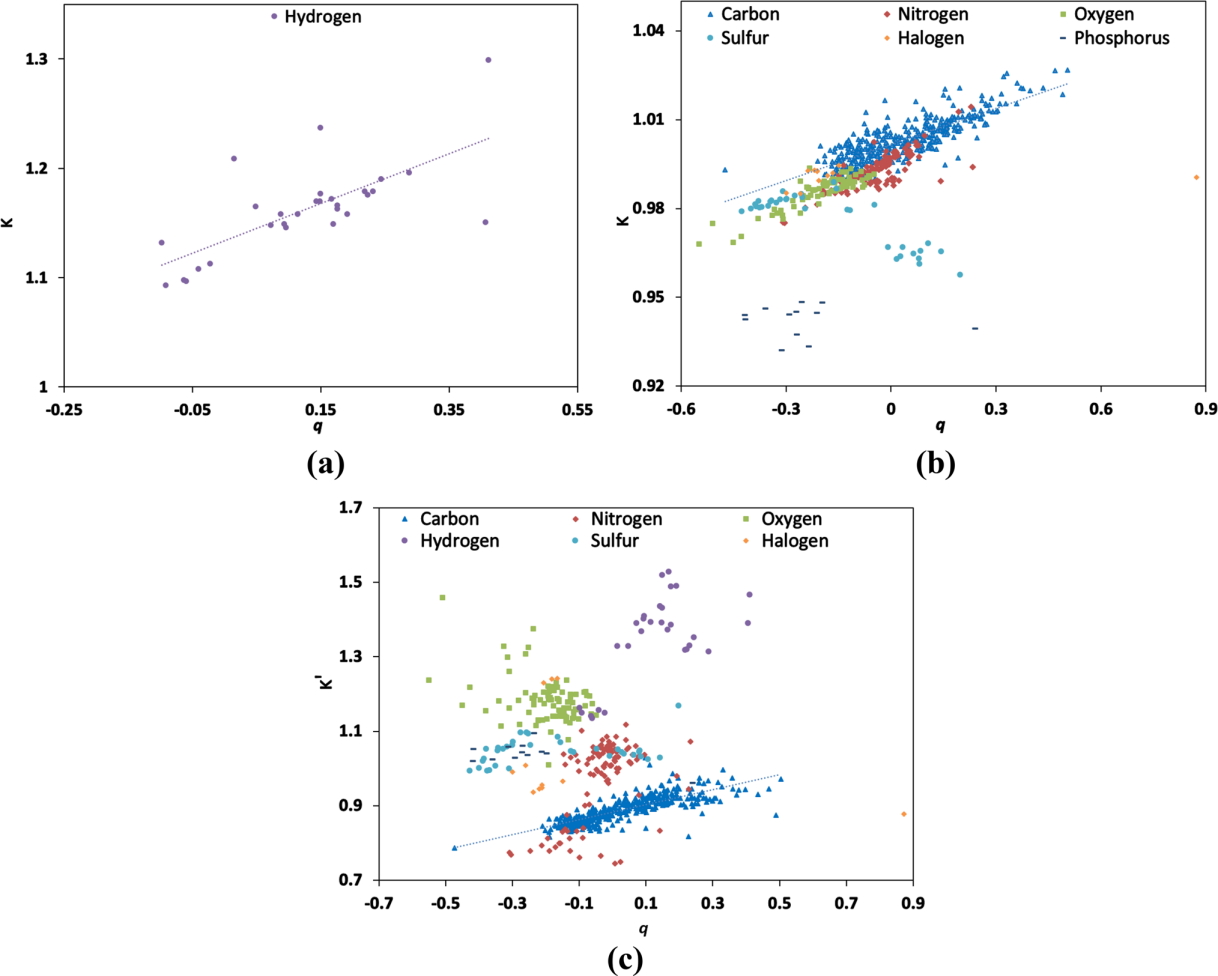
Correlation between the monopole-derived charge, *q* (*e*), and the expansion–contraction parameters (a, b) κ and (c) κ′ for hydrogen (purple circle), carbon (blue triangle), nitrogen (red diamond), oxygen (green square), sulfur (sky-blue circle), halogen (orange diamond), and phosphorus (navy-blue dash) atom types.

The earlier reports on the correlation of monopole-derived charges and expansion–contraction parameters were based on a very small number of uniform molecules and indicated a very strong correlation between the two.^57,58^ Linear dependences of κ parameters on monopole-derived charges *q* were reported with a high correlation coefficient^19^ computed on the basis of 104 atom types present in an earlier version of UBDB2006. However, in the MATTS2021 data bank, which has more diverse atom types including charged ions and atoms showing different oxidation states, the correlation between κ parameters and monopole-derived charges *q* was found to be very weak (Figure 8a). The correlation coefficient is *R*^2^ = 0.50 for all of the atom types except hydrogen atom types. The latter form a distinct group with *R*^2^ = 0.45. The deviations from linear dependency were found mostly for phosphorus and sulfur atom types. In general, the atomic charges reflect the group-electronegativity concept as defined by Huheey.^59^ However, this may not apply strictly to the atoms showing variable oxidation states including the ionic system. In the case of sulfur atom types, 11 out of 35 and, in the case of phosphorus, all 12 atom types were found to be outliers. In the case of all 11 sulfur atom types that were outliers, the central sulfur atoms had a +6 oxidation state and were connected to four neighbors with sp^3^ hybridization, while in other cases, the oxidation states of sulfur atoms were −2 and +2. In the case of phosphorus atom types, in all the cases, phosphorus atoms had a +5 oxidation state. In the case of κ′ parameters, there was no correlation found with monopole-derived charges *q* for any category of atom types except for the carbon atom types. The latter show some linear dependency (*R*^2^ = 0.60, Figure 8b).

### 3.4 Improvement of MATTS2021 in Terms of Atom and Molecule Recognition

Nearly 153 000 crystal structures of high quality containing only carbon atoms and one or more atoms of H, N, O, P, S, F, Cl, and Br element types were retrieved from the CSD. The MATTS2021 data bank has improved considerably compared to UBDB2018 in terms of percentage of atoms recognized and percentage of whole crystal structure recognized in the retrieved subset of the CSD. The recognition of the atoms, which covers now 98.13% out of more than 7 million atoms, has been improved by 1.61% points and the recognition of the whole crystal structures by 19.52% points covering now 65.64% (Table 1) of all of the structures consisting of common atom types with C, H, N, O, P, S, F, Cl, and Br atoms. The coverage is even better when the analysis is narrowed to crystal structures allowed to contain only C, H, N, or O elements (ca. 77 000 structures). 99% of individual atoms and 80% of whole structures are recognized, and only 460 (out of 651, 71%) atom types are needed.

**Table 1:** Improvement of the MATTS2021 Data Bank over UBDB 2018 in Terms of Recognized Atoms and the Whole Crystal Structures^a^ in the CSD Search Based on the Set of Molecules Obtained by Different Combinations of C, H, N, O, P, S, F, Cl, and Br Atoms

	UBDB2018	MATTS2021
recognition of atoms [%]	96.52	98.13 (98.23)
recognition of the whole crystal structures [%]	46.12	65.64 (68.15)

a Values in parentheses for MATTS2021 with Cl(−1) and Br(−1) types.

**Figure 9:**
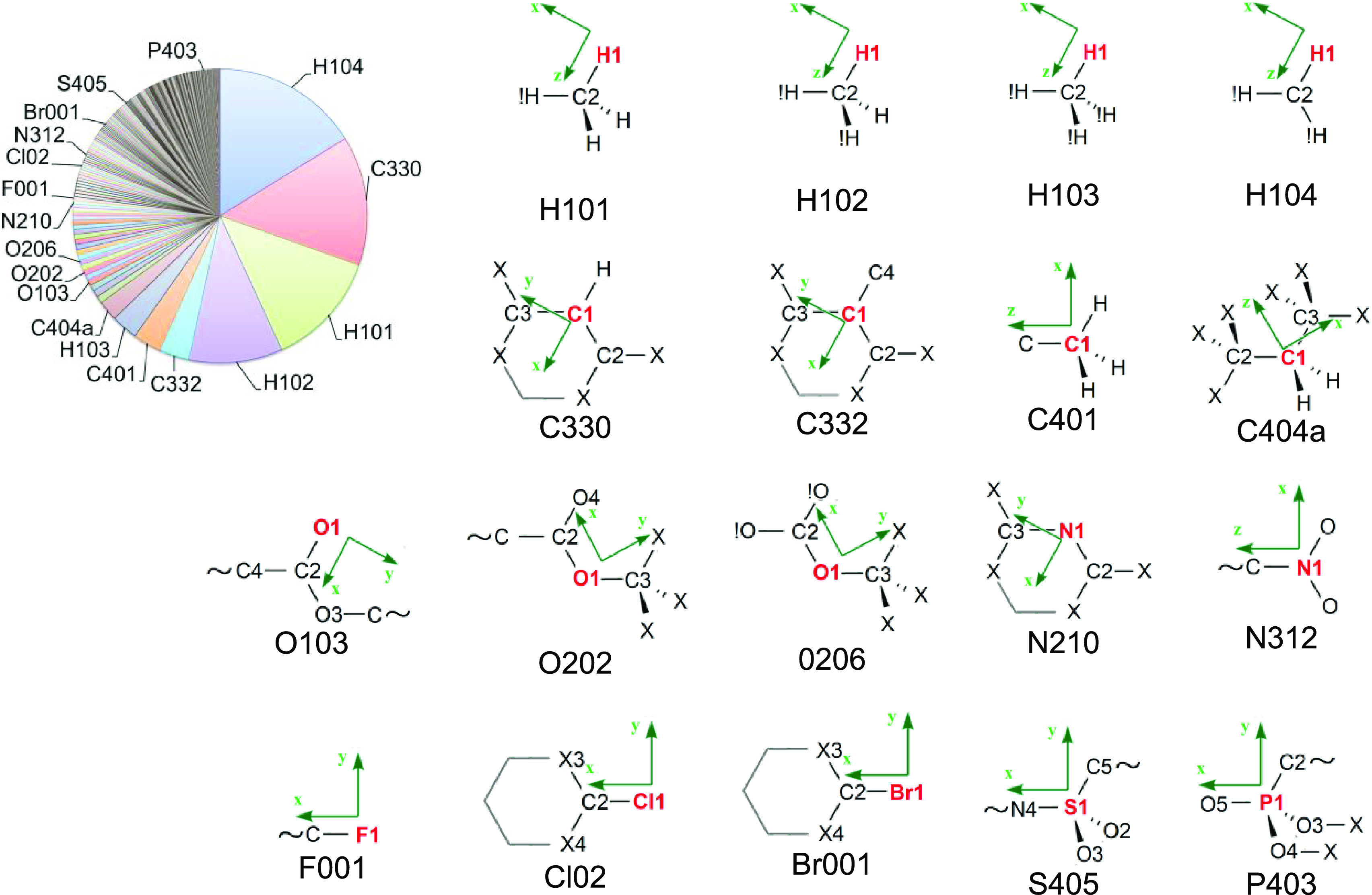
Frequency of occurrence of MATTS2021 atom types among all assigned atoms from the subset of the CSD (top left) along with schemes illustrating connectivity and local coordinate system orientations for selected atom types. For exact definitions of atom types, see the SI.

Among recognized atoms to which an atom type from the MATTS2021 data bank was assigned, the most popular are carbon and hydrogen atoms found mostly in phenyl (C330, C332, H104), methyl (C401, H101), and methylene (C404a, H1033) groups (Figure 9). These atoms cover more than 60% of all atoms. Three of the most popular oxygen types are from ester (O103, 0.63%; and O202, 0.60%) and ether (O206, 0.59%) groups, whereas the two most popular nitrogen types are from 6-membered hetero rings (N210, 0.40%) and nitro groups (N312, 0.23%). Among halogen types, the most frequent are aliphatic fluorine (F001, 0.40%) and aromatic chlorine (Cl02, 0.26%), the most frequent bromine is also aromatic (Br001, 0.18%). The most frequent sulfur type is the S405 type (0.13%, sulfur atom in a sulfonyl group connected to two terminal oxygen atoms, one carbon atom, and one nitrogen atom), and the most frequent phosphorus type is the P403 type (0.01%, phosphorus atom attached to three oxygen atoms and one carbon atom; see Figure 9).

Interestingly, the largest populations of atom types which were missing in the UBDB2018 and are now present in the MATTS2021 are aromatic carbons existing in heavily distorted aromatic rings (C3a2, 0.08%; C3a3, 0.07%; C3a1, 0.05%) and observed in such molecules like fullerenes, buckybowls, strained pyrenophanes, and others. Among the most common missing atom types, there were also fluorine and oxygen types from simple inorganic PF_6_^–^ and ClO_4_^–^ anions (F003, 0.04%; O189, 0.03%).

One of the main reasons that some atom types are still lacking is the limitation of the filtering procedure applied to remove improper structures. Using ConQuest filters while searching the CSD still resulted in many structures with clutter. The usage of ConQuest filters during the CSD search resulted in many structures with wrong geometries and disorders or missing hydrogen atoms. Some structures were further removed by custom-made additional filters, but not all. This means that the level of atom recognition with the MATTS2021 data bank is even better than that reported above.

There are also structures containing unrecognized types which constitute genuine chemical moieties like single-atom ions or ones involved in very strong hydrogen bonds. The atom type consisting of individual atoms/ions such as free chlorine, fluorine, or bromine cannot be defined by the current atom-type algorithm. Proper modeling of electron densities of such ions would require taking into account surrounding molecules, since our studies^60^ have shown that they may have charge and expansion–contraction coefficients far from the formal one. To be able to do so, the atom-typing algorithm would need to take into account not only covalent neighbors but also all other neighbors associated with the investigated atom by weak interactions. This is a cumbersome task at the moment, as it would require modeling hundreds of possible configurations and robust definitions of possible weak interactions (or acceptance of all van-der-Waals contacts). The halfway solution is to add Cl^–^ and Br^–^ atom types to the MATTS2021 data bank with *P*_val_ values following their formal charge (*q* = −1 e) and atomic scattering functions computed for isolated anions. A noticeable improvement in atom recognition can be achieved in such a way (Table 1).

The other issue that adds to missing atom types is the current limitation of the utility programs in DiSCaMB that do not properly recognize hydrogen atoms involved in strong hydrogen bonds. Due to a short H···acceptor distance, which is less than the sum of covalent radii plus the 0.4 Å threshold, the program reads them as usual covalent bonds, which results in hydrogen atoms bonded to two neighboring atoms. Again, separation of hydrogen atoms strongly hydrogen bonded from other hydrogen types is a cumbersome task for the reasons outlined above. We have made only one exception, for the H122 hydrogen type describing the middle hydrogen atom from the Zundel H_5_O_2_^+^ cation whose type was relatively easy to define. The halfway solution would be to teach the program to disregard one of the connections to hydrogen atoms, the one which is the longest, and to treat that hydrogen as any other from an analogous chemical group, not involved in strong hydrogen bonding.

There are still atom types that can be added. The largest group of atoms for which atom types are missing in the MATTS2021 data bank is nitrogen atoms in the azido group, −N_3_. There are slightly fewer than 1000 azido groups in the investigated subset of the CSD. The necessary types will be successively added in the near future. However, this will not increase much the percentage of atom type recognition, since the assignment of an atom type to 1000 atoms will increase the atom coverage by only ca. 0.01% and the whole structure coverage by only ca. 0.6% in the best case scenario.

To achieve 100% coverage, we plan to introduce two or more levels of atom type specificity (generality), each level with atom types being more and more general at the expense of a lower and lower level of multipole model parameter transferability. More general types will cover those types that are very rare, and it is not profitable to add them with atom type definitions at the same level of detail as others. Generalization of atom types will be based on some advanced statistical analysis like multivariate data clustering.^61^ The new structure of the data bank and the new software implementation facilitate a fast application of the planned developments.

## 4 Conclusions

The MATTS2021 was formed by restructuring and modifying the previous versions of UBDB which allows for a more flexible methodology in defining new atom types. The current version of MATTS2021 contains 651 atom types, among which there are 29 hydrogen, 378 carbon, 104 nitrogen, 82 oxygen, 35 sulfur, 12 phosphorus, 6 chlorine, 3 fluorine, and 2 bromine atom types. The atom types include the most general and popular atom type in the CSD along with specific atom types to cover the majority of organic small molecules, including different amino acids, peptides, nucleic acids, and most macromolecular proteins. Application of MATTS2021 on a set of 80 crystal structures including amino acids and peptides resulted in accurate and precise hydrogen atom positioning and bond distance descriptions.^25^

Apart from the aromatic five- and six-membered planar rings, three- and four-membered ring atom types were also defined which have distinguished geometry and multipole populations. Atom types describing aromatic carbons existing in heavily distorted aromatic rings were also added into MATTS2021 along with some missing two or three fused ring atom types. The atom types with most common and frequently appearing ionic systems such as ClO_4_^–^, SO_4_^2–^, PF_6_^−^, NH_4_^+^, H_3_O^+^, and the Zundel cation were also added to MATTS2021.

Averaging multipole model parameters was performed using the DiSCaMB library which allows for a manual and more flexible definition of coordinate systems, compared to LSDB used in previous versions, which adhere to the symmetry rules and can be directly stored in the data bank.

Analysis of valence population *P*_val_ and expansion–contraction parameters κ and κ′ reveals that the neighboring atom types, hybridization, geometrical strain in the ring system, and charges on the molecule impose significant impact on these parameters, especially *P*_val_ and κ′, across all of the atom types belonging to different elements in MATTS2021. The influence of charges on molecules in the ionic system were more prominent in all of the cases, and their *P*_val_ and κ′ parameters differ most in a particular subgroup of atom types. The κ parameter was found to be more stable with the changes in neighboring atom types, hybridization, geometrical strain in the ring system, and charges on the molecule. Some other factors must influence the variability of the κ parameter.

Contrary to what was seen before, the monopole-derived charges correlate to κ parameters only in those systems showing lower oxidation states. The third row element belonging to groups 5 and 6 such as phosphorus and sulfur which can have higher oxidation states of +5 and +6, respectively, deviates significantly. Also, the ionic systems were majorly found to be outliers. There were no correlations found between monopole-derived charges and κ′ parameters except in the case of carbon.

The recognition of the atoms, which now covers 98.17%, has been improved by 1.61% and that of the whole crystal structures by 18.45% which now covers 66.76% of all of the structures consisting of C, N, O, H, P, S, F, Cl, and/or Br atoms, compared to UBDB2018. Structures having a very strong intra-/intermolecular hydrogen bond with very short distances, which do not follow the criteria set for noncovalent bond lengths, cannot be included in the present data bank.

Further application of MATTS2021 will be explored for refinement of electron diffraction data which is possible with the earlier version of UBDB.^41,62,63^ With the addition of the diverse yet most common atom types in MATTS2021, it will be possible to evaluate the electrostatic interactions in the screening of organic small molecules and macromolecular structures accurately and quickly.^33^ MATTS2021 can be applied in drug discovery and docking studies as it enhances the physical meaning of molecular electrostatic potential descriptors used to construct predictive quantitative structure–activity relationship/quantitative structure–property relationship (QSAR/QSPR) models.^21^

More statistics on the clustering of multipoles and their variations across different atom types will be presented in a future article.^61^

## Supporting Information Available

Details of each term in the data bank entry, details about the local coordinate system assignment, most popular atom type entries mentioned in Figure 9, and CSD refcodes of all model molecules used for creating the MATTS2021 data bank (PDF)

## Notes

*Data and Software Availability*. Starting coordinates of model molecules used to create the MATTS2021 data bank can be retrieved from the CSD (https://www.ccdc.cam.ac.uk) using the refcodes given in the SI. The current version of the data bank, MATTS2021, can be printed out from one of the DiSCaMB application programs available for download free of charge for nonprofit users from http://4xeden.uw.edu.pl/software. The source code for the DiSCaMB library is available for download free of charge under the MIT license from http://4xeden.uw.edu.pl/software. Local programs and scripts necessary to reproduce the results published in this paper are available from the authors upon request.
