# Supplementary material for: Multipolar Atom Types from Theory and Statistical Clustering (MATTS) Data Bank: Restructurization and Extension of UBDB
Source: J Chem Inf Model. 2022 Aug 9;62(16):3752–65. doi: 10.1021/acs.jcim.2c00144 (PMC9400107; doi:10.1021/acs.jcim.2c00144)
Supplement: Supplementary file 1 — ci2c00144_si_001.pdf [file ci2c00144_si_001.pdf]

Supporting Information

Multipolar Atom Types from Theory and Statistical clustering (MATTS) Databank. Restructurization and extension of UBDB

Kunal Kumar Jha, Barbara Gruza, Aleksandra Sypko, Prashant Kumar, Michał Leszek Chodkiewicz, Paulina Maria Dominiak**

Biological and Chemical Research Centre, Department of Chemistry, University of Warsaw, ul. Żwirki i Wigury 101, 02-089, Warszawa, Poland

*Correspondence e-mail: mchodkiewicz@chem.uw.edu.pl, pdomin@chem.uw.edu.pl

Format Description of the Entry in Databank

Each atom type is described in a separate databank entry which starts with the "ENTRY" keyword followed by a unique identification code "ID" field. Next a "COMMENT" line is printed, currently documenting whether the entry is "NEW" or from "UBDB 2018". After that, the number of instances (NOI) of model atoms used to compute mean values of multipole model parameters while parametrizing the entry is given.

The next section defines atom type topology by providing "ATOM DESCRIPTORS" for the "central atom" and often for "1st", "2nd" or even "3rd neighbours". Each central atom and neighbour atom (if specified) is described by the following descriptor fields:

(1) unique atom label, in which the first letter(s) code(s) for element type of the atom.

(2) "CONNECTED_TO" characterizing atom connectivity by providing a list of atoms connected to it. The connected atoms (neighbours) can be specified by (a) a unique atom label, (b) chemical element symbol (if later there is no separate descriptors line for that neighbour), (c) element symbol preceded by '!' which indicates that the neighbour is of any element except of that indicated one, (d) 'X' which indicates atom of any element, (e) '*' which indicates any number of neighbours of any element type including zero, and by (f) '-' meaning the central atom has no neighbours. Atoms closer than the sum of their respective covalent radii[1] plus covalent bond threshold are recognized as connected. The value of 0.4 Å was set for that threshold while constructing the MATTS2021 databank.

(3) group "PLANARITY" which is defined for atoms having at least 3 first neighbours and is specified with '+', '-' or '*' sign indicating whether the atom and its first neighbours are planar, non-planar or have any/undefined planarity, respectively. The planarity is evaluated on the basis of planarity indexed p computed for the set of n atomic positions and defined as: $p = \frac{1}{n-3}\sqrt{\sum_k d_k^2}$, where d_k is a distance of k-th atom from the plane which minimizes the planarity index. The applied procedure is based on methods described by Haneef et al.[2] and Urzhumtsev[3]. Atom planarity threshold is associated with this descriptor, which was set to 0.1 Å while constructing the MATTS2021 databank.

(4) “PLANAR_RING_WITH_PLANAR_ATOMS” defining if the atom belongs or not to a planar ring. Definition of a planar ring has been modified from the one previously used for constructing UBDB[4–6]. In MATTS2021 databank only planar rings are considered in which each member has max. 3 first neighbours and belongs to planar group. Previously, number of first neighbours and group planarity was not verified, what resulted in mixing truly aromatic rings with highly strained but not aromatic rings, like in oxocyclopenten or oxazolidinone moieties. Characters are used to label the rings, eg.: 6A/6B/5A/5. This labelling allows to indicate if atoms belong to the same (e.g. by specifying 6A for both atoms) or to a different rings (e.g. by specifying 6A for one atom and 6B for another one). To specify that the atom belongs to many rings, the labels of that rings are listed (e.g. 6A,5A or 6A,6B). It is also possible to specify rings without labelling them by declaring their size only (e.g. 5). It is also possible to indicate that the atom does not belong to some specific ring by posting the “!” in front of that ring label (e.g. !6A). If atom does not belong to any ring, it is indicated with '-' character. The '*' character indicates that ring membership is not defined or belongingness to other than already labelled rings is allowed (e.g. “6A,*” means that atom belongs to one or more rings and one of them is the 6A ring). '+' means the atom belongs to any planar ring (one or more). Setting used for perception of planar rings include the thresholds for ring planarity and for atom-in-ring planarity. Value of 0.1 Å was used for both thresholds while constructing the MATTS2021 databank. Only rings of size between 3 and 8 members are considered.

(5) independently from the (4) descriptor, new descriptors, “IN_3_MEMBER_RING” and “IN_4_MEMBER_RING”, not used previously in the UBDB, are added. Here 3- or 4-membered rings are considered, regardless if they are planar or not, or if their members belong to planar groups or not. Atoms belonging to 3- or 4-membered rings have strained geometry, and in

consequence their valence electrons may be differently distributed in space compared to unrestrained atoms.

The next section contains information about "LOCAL COORDINATE SYSTEM" in which multipole functions are expressed, and local "SYMMETRY" which has to be fulfilled by multipole model parameters stored in the databank. In case of chiral atom types, additional field "CHIRALITY" appear, where the list of three first-neighbours defining the handedness of the group is given. Chirality is defined locally, that is, only by the character of the nearest neighbours. More details about this section are given in the subchapter 2.2.

The last section contains "MULTIPOLE MODEL PARAMETERS" preceded by a "PARAMETER MODIFICATION DATE" field. The parameters are derived by computing mean values from a set of atoms retrieved from model molecules and recognized as belonging to a given atom type. Along with the mean values, sample standard deviations ($ssd = \sqrt{\sum(x - \acute{x})^2/(n-1)}$) are stored for each parameter. Only these P_{lm} parameters are stored in the databank which are: (a) larger than 0.002 e, (b) larger than their one standard deviation, (c) allowed to be different from zero by the symmetry defined in the "SYMMETRY" field.

Local Coordinate System Assignment

Pseudoatom electron density functions are expressed using the local Cartesian coordinate system. The Cartesian system is centred on the central atom of the atom type and oriented according to neighbouring atoms. The local coordinate system is defined in the XD style[7] "ax1 p1 ax2 p2 R/L", where ax1 and ax2 defines the type of axis (X or Y or Z) and p1 and p2 defines directions. The first axis (**ax1**) is oriented along the direction vector defined by **p1**. The second axis **ax2** lays on a plane defined by **p1** and **p2** directions and is as close as to **p2**, i.e. **ax2** is directed along (**p1** x **p2**)

x **p1**. The third axis (**ax3**) is perpendicular to the two previous axes **ax3** = h **(ax1** x **ax2)**, where h is 1 or -1 and it is chosen in such a way that the required handedness of the coordinate system (R/L) is achieved. Each direction (p1 and p2) can be specified in the MATTS2021 databank by the following ways:

(1) unique atom label (e.g. C1) - direction from the central atom to point located at atom (C1),

(2) chemical element symbol (atom label) (e.g. N(C1)) - direction from the central atom to point located at an atom of element given by atom symbol bonded to atom specified in brackets (in the example any nitrogen atom connected to atom C1),

(3) r(ring label) (e.g. r(6A)) - direction from the central atom to point in the centre of ring (6A),

(4) average_direction(atom_label_1,..,atom_label_n) (e.g. average_direction(C1,C2)) - average direction from the central atom to atoms on the list (also element symbols instead of atom label can be used e.g. average direction (N,N) will be an average direction of vectors from central atom to two nitrogen atoms which are connected to the central atom). For n neighbours of the central atoms the average direction is defined as follows:

$$\boldsymbol{r} = \frac{1}{n}\sum_{k=1}^{k=n}\frac{\boldsymbol{r}_k - \boldsymbol{r}_c}{\|\boldsymbol{r}_k - \boldsymbol{r}_c\|}$$

where $\boldsymbol{r}_k$ is a position of the k-th neighbor, $\boldsymbol{r}_c$ is a central atom position and $\|\boldsymbol{v}\|$ denotes length of a vector $\boldsymbol{v}$.

(5) any_atom(atom_label) (e.g. any_atom(C1)) - direction from the central atom to any atom bonded to atom specified with atom label,

(6) !chemical element symbol(atom label) - direction from the central atom to any atom of different chemical element than given by chemical element symbol bonded to atom specified with atom label (e.g. !H(C1) - any non-hydrogen bonded to C1),

(7) any_orthogonal - any_orthogonal - any direction orthogonal to the other direction used in the definition of the local coordinate system. For example in the following definition of local coordinate system:

Z P2 Y any_orthogonal R

the Z direction is defined by a vector $\mathbf{v}_z$ from the central atom to the atom P2 and the Y direction is chosen as a direction orthogonal to $\mathbf{v}_z$. In practice it is calculated in the following way:

1. Cross products of $\mathbf{v}_z$ with $\mathbf{x}$=[1,0,0] and $\mathbf{y}$=[0,1,0] are calculated:

$$\mathbf{c}_x = \mathbf{v}_z \times \mathbf{x}$$

$$\mathbf{c}_y = \mathbf{v}_z \times \mathbf{y}$$

2. The one of the two vectors ($\mathbf{c}_x$ and $\mathbf{c}_y$) with higher norm (i.e. more orthogonal to $\mathbf{v}_z$) is chosen as the Y direction.

In the case when both directions in the local coordinate system definition are defined as any_orthogonal then the local coordinate system corresponds to the global coordinate system (i.e. **x**=[1,0,0], **y**=[0,1,0], **z**= [0,0,1], where the **x**-axis is collinear with **a**-axis and **z**-axis is parallel to **c***-axis.).

For the following cases: average_direction (element symbol, element symbol), atom symbol (atom label), any_atom(atom_label) and !chemical element symbol(atom label) it is possible that the atom to be used in the definition has to be chosen from a group of atoms fulfilling requirements

given by specification – e.g. for !H(C1) - any non-hydrogen bonded to C1 – there may be many non-hydrogen atoms bonded to C1. In such a situation an atom with the highest atomic number is chosen, if there is more than one then the atom with the lowest valence is chosen and if still there is no unique choice than the atom with shortest bond to the named atom is chosen (to the central atom in the case of average_direction). No dummy atoms are explicitly defined in local coordinate system definitions in the MATTS2021 databank, instead average directions from explicitly specified neighbouring atoms are computed on demand. The right-handed (R) coordinate system is always defined in the atom type entry. In case of chiral atom types, the databank creation program and the databank application programs takes care to inverse multipole model parameters or to inverse coordinate system for atoms which are recognised as the other enantiomer.

Scheme S1. Entries in the MATTS2021 databank for the most popular atom types presented in Figure 9.

```
ENTRY
  ID
    H101
  COMMENT
    in UBDB2018: H101 KJ
  NOI
    10089
  ATOM DESCRIPTORS
#  central atom
    H1    CONNECTED_TO  C2               PLANARITY *  PLANAR_RING_WITH_PLANAR_ATOMS -        IN_3_MEMBER_RING - IN_4_MEMBER_RING -
#  1-st neighbors
    C2    CONNECTED_TO  H1,H,H,!H        PLANARITY *  PLANAR_RING_WITH_PLANAR_ATOMS -        IN_3_MEMBER_RING * IN_4_MEMBER_RING *
  LOCAL COORDINATE SYSTEM
    Z C2 X !H(C2) R
  SYMMETRY
    cyl
  PARAMETER MODIFICATION DATE
    Fri Dec  3 10:28:39 2021
  MULTIPOLE MODEL PARAMETERS
    PVAL          1.092(38) KAPPA         1.093(13) KPRIM         1.149(19)
    PLMS   1  0     0.186(10) PLMS   2  0    0.0857(87)
```

```
ENTRY
  ID
    H102
  COMMENT
    in UBDB2018: H102 KJ
  NOI
    7124
  ATOM DESCRIPTORS
#   central atom
    H1     CONNECTED_TO  C2              PLANARITY *  PLANAR_RING_WITH_PLANAR_ATOMS -        IN_3_MEMBER_RING - IN_4_MEMBER_RING -
#   1-st neighbors
    C2     CONNECTED_TO  H1,H,!H,!H      PLANARITY *  PLANAR_RING_WITH_PLANAR_ATOMS *        IN_3_MEMBER_RING * IN_4_MEMBER_RING *
  LOCAL COORDINATE SYSTEM
    Z C2 X !H(C2) R
  SYMMETRY
    cyl
  PARAMETER MODIFICATION DATE
    Fri Dec  3 10:28:39 2021
  MULTIPOLE MODEL PARAMETERS
    PVAL          1.064(45) KAPPA        1.098(16) KPRIM        1.140(31)
    PLMS   1  0     0.180(15) PLMS   2  0     0.086(11)

ENTRY
  ID
    H103
  COMMENT
    in UBDB2018: H103 KJ
  NOI
    1962
  ATOM DESCRIPTORS
#   central atom
    H1     CONNECTED_TO  C2              PLANARITY *  PLANAR_RING_WITH_PLANAR_ATOMS -        IN_3_MEMBER_RING - IN_4_MEMBER_RING -
#   1-st neighbors
    C2     CONNECTED_TO  H1,!H,!H,!H     PLANARITY *  PLANAR_RING_WITH_PLANAR_ATOMS *        IN_3_MEMBER_RING * IN_4_MEMBER_RING *
  LOCAL COORDINATE SYSTEM
    Z C2 X !H(C2) R
  SYMMETRY
    cyl
  PARAMETER MODIFICATION DATE
    Fri Dec  3 10:28:39 2021
  MULTIPOLE MODEL PARAMETERS
    PVAL          1.023(59) KAPPA        1.113(22) KPRIM        1.150(48)
    PLMS   1  0     0.171(23) PLMS   2  0     0.083(18)

ENTRY
  ID
    H104
  COMMENT
    in UBDB2018: H104 KJ
  NOI
    11700
  ATOM DESCRIPTORS
#   central atom
    H1     CONNECTED_TO  C2              PLANARITY *  PLANAR_RING_WITH_PLANAR_ATOMS -        IN_3_MEMBER_RING - IN_4_MEMBER_RING -
#   1-st neighbors
    C2     CONNECTED_TO  H1,!H,!H        PLANARITY *  PLANAR_RING_WITH_PLANAR_ATOMS *        IN_3_MEMBER_RING * IN_4_MEMBER_RING *
  LOCAL COORDINATE SYSTEM
    Z C2 X !H(C2) R
  SYMMETRY
    cyl
  PARAMETER MODIFICATION DATE
    Fri Dec  3 10:28:39 2021
  MULTIPOLE MODEL PARAMETERS
    PVAL          1.041(37) KAPPA        1.108(13) KPRIM        1.156(24)
    PLMS   1  0     0.181(12) PLMS   2  0    0.0846(98)
```

```
ENTRY
  ID
    C330
  COMMENT
    in UBDB2018: C330 KJ
  NOI
    10017
  ATOM DESCRIPTORS
#   central atom
    C1    CONNECTED_TO  C2,C3,H           PLANARITY +  PLANAR_RING_WITH_PLANAR_ATOMS 6A       IN_3_MEMBER_RING - IN_4_MEMBER_RING -
#   1-st neighbors
    C2    CONNECTED_TO  C1,X,X            PLANARITY +  PLANAR_RING_WITH_PLANAR_ATOMS 6A,*     IN_3_MEMBER_RING * IN_4_MEMBER_RING *
    C3    CONNECTED_TO  C1,X,X            PLANARITY +  PLANAR_RING_WITH_PLANAR_ATOMS 6A,*     IN_3_MEMBER_RING * IN_4_MEMBER_RING *
  LOCAL COORDINATE SYSTEM
    X r(6A) Y C2 R
  SYMMETRY
    m
  PARAMETER MODIFICATION DATE
    Fri Dec  3 10:28:39 2021
  MULTIPOLE MODEL PARAMETERS
    PVAL          3.910(48) KAPPA       1.0033(33) KPRIM        0.909(14)
    PLMS    1  1     0.055(16) PLMS    2  0    -0.179(23) PLMS    2  2    -0.028(13) PLMS    3  1    0.0299(65)
    PLMS    3  3    -0.263(20) PLMS    4  0    0.0202(53) PLMS    4  2   -0.0196(55)

ENTRY
  ID
    C332
  COMMENT
    in UBDB2018: C332 KJ
  NOI
    2012
  ATOM DESCRIPTORS
#   central atom
    C1    CONNECTED_TO  C2,C3,C4          PLANARITY +  PLANAR_RING_WITH_PLANAR_ATOMS 6A       IN_3_MEMBER_RING - IN_4_MEMBER_RING -
#   1-st neighbors
    C2    CONNECTED_TO  C1,X,X            PLANARITY +  PLANAR_RING_WITH_PLANAR_ATOMS 6A,*     IN_3_MEMBER_RING * IN_4_MEMBER_RING *
    C3    CONNECTED_TO  C1,X,X            PLANARITY +  PLANAR_RING_WITH_PLANAR_ATOMS 6A,*     IN_3_MEMBER_RING * IN_4_MEMBER_RING *
    C4    CONNECTED_TO  C1,*              PLANARITY *  PLANAR_RING_WITH_PLANAR_ATOMS !6A      IN_3_MEMBER_RING * IN_4_MEMBER_RING *
  LOCAL COORDINATE SYSTEM
    X r(6A) Y C2 R
  SYMMETRY
    m
  PARAMETER MODIFICATION DATE
    Fri Dec  3 10:28:39 2021
  MULTIPOLE MODEL PARAMETERS
    PVAL          4.027(83) KAPPA       0.9996(54) KPRIM        0.885(22)
    PLMS    1  1     0.050(23) PLMS    2  0    -0.181(34) PLMS    2  2    -0.036(18) PLMS    3  1    0.0253(98)
    PLMS    3  3    -0.288(29) PLMS    4  0    0.0187(83) PLMS    4  2   -0.0134(97)

ENTRY
  ID
    C401
  COMMENT
    in UBDB2018: C401 KJ
  NOI
    2344
  ATOM DESCRIPTORS
#   central atom
    C1    CONNECTED_TO  C,H,H,H           PLANARITY -  PLANAR_RING_WITH_PLANAR_ATOMS -        IN_3_MEMBER_RING - IN_4_MEMBER_RING -
  LOCAL COORDINATE SYSTEM
    Z C(C1) X H(C1) R
  SYMMETRY
    3m
  PARAMETER MODIFICATION DATE
    Fri Dec  3 10:28:39 2021
  MULTIPOLE MODEL PARAMETERS
    PVAL          3.565(83) KAPPA       1.0208(55) KPRIM        0.931(16)
    PLMS    2  0    -0.017(14) PLMS    3  0     0.232(19) PLMS    3  3     0.187(21) PLMS    4  0     0.045(12)
    PLMS    4  3    -0.079(12)
```

```
ENTRY
  ID
    C404a
  COMMENT
    in UBDB2018: C404 splitted KJ
  NOI
    1300
  ATOM DESCRIPTORS
#  central atom
    C1     CONNECTED_TO  C2,C3,H,H          PLANARITY -  PLANAR_RING_WITH_PLANAR_ATOMS -        IN_3_MEMBER_RING - IN_4_MEMBER_RING -
#  1-st neighbors
    C2     CONNECTED_TO  C1,X,X,X           PLANARITY -  PLANAR_RING_WITH_PLANAR_ATOMS -        IN_3_MEMBER_RING * IN_4_MEMBER_RING *
    C3     CONNECTED_TO  C1,X,X,X           PLANARITY -  PLANAR_RING_WITH_PLANAR_ATOMS -        IN_3_MEMBER_RING * IN_4_MEMBER_RING *
  LOCAL COORDINATE SYSTEM
    Z average_direction(C2,C3) X H(C1) R
  SYMMETRY
    mm2
  PARAMETER MODIFICATION DATE
    Fri Dec  3 10:28:39 2021
  MULTIPOLE MODEL PARAMETERS
    PVAL          3.762(83) KAPPA        1.0080(50) KPRIM        0.906(23)
    PLMS    1  0      0.027(18) PLMS    3  2    -0.318(51) PLMS    4  0     -0.084(18) PLMS    4  4      0.061(14)

ENTRY
  ID
    O103
  COMMENT
    in UBDB2018: O103 KJ
  NOI
    440
  ATOM DESCRIPTORS
#  central atom
    O1     CONNECTED_TO  C2                 PLANARITY *  PLANAR_RING_WITH_PLANAR_ATOMS -        IN_3_MEMBER_RING - IN_4_MEMBER_RING -
#  1-st neighbors
    C2     CONNECTED_TO  O1,O3,C4           PLANARITY +  PLANAR_RING_WITH_PLANAR_ATOMS -        IN_3_MEMBER_RING * IN_4_MEMBER_RING *
#  2-nd neighbors
    O3     CONNECTED_TO  C2,C               PLANARITY *  PLANAR_RING_WITH_PLANAR_ATOMS *        IN_3_MEMBER_RING * IN_4_MEMBER_RING *
    C4     CONNECTED_TO  C2,*               PLANARITY *  PLANAR_RING_WITH_PLANAR_ATOMS *        IN_3_MEMBER_RING * IN_4_MEMBER_RING *
  LOCAL COORDINATE SYSTEM
    X C2 Y O3 R
  SYMMETRY
    m
  PARAMETER MODIFICATION DATE
    Fri Dec  3 10:28:39 2021
  MULTIPOLE MODEL PARAMETERS
    PVAL          6.112(34) KAPPA        0.9885(17) KPRIM        1.160(18)
    PLMS    1 -1    -0.0044(33) PLMS    1  1    -0.0978(42) PLMS    2  0    -0.0736(87) PLMS    2  2    -0.0685(49)
    PLMS    3  1    -0.0048(30) PLMS    3  3     0.0149(38) PLMS    4  0    -0.0058(23) PLMS    4  2     0.0033(22)
    PLMS    4  4     0.0042(28)

ENTRY
  ID
    O202
  COMMENT
    in UBDB2018: O202 splitted KJ
  NOI
    387
  ATOM DESCRIPTORS
#  central atom
    O1     CONNECTED_TO  C2,C3              PLANARITY +  PLANAR_RING_WITH_PLANAR_ATOMS -        IN_3_MEMBER_RING - IN_4_MEMBER_RING -
#  1-st neighbors
    C2     CONNECTED_TO  O1,O4,C            PLANARITY +  PLANAR_RING_WITH_PLANAR_ATOMS -        IN_3_MEMBER_RING * IN_4_MEMBER_RING *
    C3     CONNECTED_TO  O1,X,X,X           PLANARITY -  PLANAR_RING_WITH_PLANAR_ATOMS *        IN_3_MEMBER_RING * IN_4_MEMBER_RING *
#  2-nd neighbors
    O4     CONNECTED_TO  C2                 PLANARITY *  PLANAR_RING_WITH_PLANAR_ATOMS -        IN_3_MEMBER_RING * IN_4_MEMBER_RING *
  LOCAL COORDINATE SYSTEM
    X C2 Y C3 R
  SYMMETRY
    m
  PARAMETER MODIFICATION DATE
    Fri Dec  3 10:28:39 2021
  MULTIPOLE MODEL PARAMETERS
    PVAL          6.192(22) KAPPA        0.9879(11) KPRIM        1.186(24)
    PLMS    1 -1    -0.0765(53) PLMS    1  1    -0.0467(60) PLMS    2 -2     0.0631(48) PLMS    2  0     0.0629(63)
    PLMS    2  2    -0.0210(62) PLMS    3 -1    -0.0151(31) PLMS    3  3     0.0392(51) PLMS    4 -4     0.0114(45)
    PLMS    4 -2     0.0043(19) PLMS    4  0     0.0071(22) PLMS    4  4     0.0087(33)
```

```
ENTRY
  ID
    O206
  COMMENT
    in UBDB2018: O206 KJ
  NOI
    350
  ATOM DESCRIPTORS
#  central atom
    O1    CONNECTED_TO  C2,C3             PLANARITY +  PLANAR_RING_WITH_PLANAR_ATOMS -       IN_3_MEMBER_RING - IN_4_MEMBER_RING -
#  1-st neighbors
    C2    CONNECTED_TO  O1,!O,!O          PLANARITY +  PLANAR_RING_WITH_PLANAR_ATOMS *       IN_3_MEMBER_RING * IN_4_MEMBER_RING *
    C3    CONNECTED_TO  O1,X,X,X          PLANARITY -  PLANAR_RING_WITH_PLANAR_ATOMS -       IN_3_MEMBER_RING * IN_4_MEMBER_RING *
  LOCAL COORDINATE SYSTEM
    X C2 Y C3 R
  SYMMETRY
    m
  PARAMETER MODIFICATION DATE
    Fri Dec  3 10:28:39 2021
  MULTIPOLE MODEL PARAMETERS
    PVAL          6.217(27) KAPPA        0.9864(14) KPRIM        1.184(22)
    PLMS   1 -1   -0.0839(73) PLMS   1  1   -0.0535(45) PLMS   2 -2    0.0582(31) PLMS   2  0     0.091(11)
    PLMS   2  2   -0.0290(68) PLMS   3 -1   -0.0138(26) PLMS   3  1   -0.0061(28) PLMS   3  3    0.0393(56)
    PLMS   4 -4    0.0122(31) PLMS   4 -2    0.0038(15) PLMS   4  0    0.0080(18) PLMS   4  4    0.0087(27)

ENTRY
  ID
    N210
  COMMENT
    in UBDB2018: N210 KJ
  NOI
    324
  ATOM DESCRIPTORS
#  central atom
    N1    CONNECTED_TO  C2,C3             PLANARITY +  PLANAR_RING_WITH_PLANAR_ATOMS 6A      IN_3_MEMBER_RING - IN_4_MEMBER_RING -
#  1-st neighbors
    C2    CONNECTED_TO  N1,X,X            PLANARITY +  PLANAR_RING_WITH_PLANAR_ATOMS 6A,*    IN_3_MEMBER_RING * IN_4_MEMBER_RING *
    C3    CONNECTED_TO  N1,X,X            PLANARITY +  PLANAR_RING_WITH_PLANAR_ATOMS 6A,*    IN_3_MEMBER_RING * IN_4_MEMBER_RING *
  LOCAL COORDINATE SYSTEM
    X r(6A) Y C2 R
  SYMMETRY
    m
  PARAMETER MODIFICATION DATE
    Fri Dec  3 10:28:39 2021
  MULTIPOLE MODEL PARAMETERS
    PVAL          5.010(51) KAPPA        0.9895(29) KPRIM        1.044(21)
    PLMS   1  1   -0.1347(86) PLMS   2  0    -0.098(34) PLMS   2  2    0.0781(51) PLMS   3  1   -0.0232(35)
    PLMS   3  3   -0.0760(80) PLMS   4  0   -0.0043(31) PLMS   4  4   -0.0205(58)

ENTRY
  ID
    N312
  COMMENT
    in UBDB2018: N312 KJ
  NOI
    231
  ATOM DESCRIPTORS
#  central atom
    N1    CONNECTED_TO  O,O,C             PLANARITY +  PLANAR_RING_WITH_PLANAR_ATOMS -       IN_3_MEMBER_RING - IN_4_MEMBER_RING -
  LOCAL COORDINATE SYSTEM
    Z C(N1) X O(N1) R
  SYMMETRY
    mm2
  PARAMETER MODIFICATION DATE
    Fri Dec  3 10:28:39 2021
  MULTIPOLE MODEL PARAMETERS
    PVAL          5.035(24) KAPPA        0.9865(20) KPRIM       0.7651(99)
    PLMS   1  0     0.023(18) PLMS   2  0     0.082(17) PLMS   2  2     0.198(14) PLMS   3  0     0.350(13)
    PLMS   3  2    -0.268(12) PLMS   4  0     0.040(12) PLMS   4  2     0.013(11) PLMS   4  4     0.057(10)
```

```
ENTRY
  ID
    F001
  COMMENT
    in UBDB2018: F001 KJ
  NOI
    338
  ATOM DESCRIPTORS
#  central atom
    F1    CONNECTED_TO  C2              PLANARITY *  PLANAR_RING_WITH_PLANAR_ATOMS -       IN_3_MEMBER_RING - IN_4_MEMBER_RING -
#  1-st neighbors
    C2    CONNECTED_TO  F1,*            PLANARITY *  PLANAR_RING_WITH_PLANAR_ATOMS -       IN_3_MEMBER_RING * IN_4_MEMBER_RING *
  LOCAL COORDINATE SYSTEM
    X C2 Y any_orthogonal R
  SYMMETRY
    m
  PARAMETER MODIFICATION DATE
    Fri Dec  3 10:28:39 2021
  MULTIPOLE MODEL PARAMETERS
    PVAL          7.166(32) KAPPA       0.9920(18) KPRIM        1.242(35)
    PLMS   1  1   -0.0636(30) PLMS   2  0    0.0540(50) PLMS   2  2   -0.0886(62) PLMS   3  1   -0.0077(23)
    PLMS   3  3    0.0095(24) PLMS   4  0    0.0036(13) PLMS   4  2   -0.0052(10) PLMS   4  4    0.0064(11)

ENTRY
  ID
    Cl02
  COMMENT
    in UBDB2018: Cl02 KJ
  NOI
    144
  ATOM DESCRIPTORS
#  central atom
    Cl1   CONNECTED_TO  C2              PLANARITY *  PLANAR_RING_WITH_PLANAR_ATOMS -       IN_3_MEMBER_RING - IN_4_MEMBER_RING -
#  1-st neighbors
    C2    CONNECTED_TO  Cl1,X3,X4       PLANARITY +  PLANAR_RING_WITH_PLANAR_ATOMS 6A      IN_3_MEMBER_RING * IN_4_MEMBER_RING *
#  2-nd neighbors
    X3    CONNECTED_TO  C2,*            PLANARITY +  PLANAR_RING_WITH_PLANAR_ATOMS 6A,*    IN_3_MEMBER_RING * IN_4_MEMBER_RING *
    X4    CONNECTED_TO  C2,*            PLANARITY +  PLANAR_RING_WITH_PLANAR_ATOMS 6A,*    IN_3_MEMBER_RING * IN_4_MEMBER_RING *
  LOCAL COORDINATE SYSTEM
    X C2 Y X3 R
  SYMMETRY
    m
  PARAMETER MODIFICATION DATE
    Fri Dec  3 10:28:39 2021
  MULTIPOLE MODEL PARAMETERS
    PVAL          7.212(40) KAPPA       0.9928(15) KPRIM        0.948(21)
    PLMS   1  1   -0.0416(93) PLMS   2  0    0.0742(64) PLMS   2  2   -0.1579(68) PLMS   3  1   -0.0568(61)
    PLMS   3  3    0.0688(56) PLMS   4  0    0.0099(23) PLMS   4  2   -0.0155(26) PLMS   4  4    0.0185(32)

ENTRY
  ID
    Br001
  COMMENT
    UBDB2018: KJ
  NOI
    12
  ATOM DESCRIPTORS
#  central atom
    Br1   CONNECTED_TO  C2              PLANARITY *  PLANAR_RING_WITH_PLANAR_ATOMS -       IN_3_MEMBER_RING - IN_4_MEMBER_RING -
#  1-st neighbors
    C2    CONNECTED_TO  Br1,X3,X4       PLANARITY +  PLANAR_RING_WITH_PLANAR_ATOMS 6A      IN_3_MEMBER_RING - IN_4_MEMBER_RING -
#  2-nd neighbors
    X3    CONNECTED_TO  C2,*            PLANARITY +  PLANAR_RING_WITH_PLANAR_ATOMS 6A,*    IN_3_MEMBER_RING * IN_4_MEMBER_RING *
    X4    CONNECTED_TO  C2,*            PLANARITY +  PLANAR_RING_WITH_PLANAR_ATOMS 6A,*    IN_3_MEMBER_RING * IN_4_MEMBER_RING *
  LOCAL COORDINATE SYSTEM
    X C2 Y X3 R
  SYMMETRY
    m
  PARAMETER MODIFICATION DATE
    Fri Dec  3 10:28:39 2021
  MULTIPOLE MODEL PARAMETERS
    PVAL          7.26(15) KAPPA        0.9850(24) KPRIM        1.009(15)
    PLMS   1  1    -0.078(10) PLMS   2  0    0.0941(93) PLMS   2  2    -0.173(11) PLMS   3  1   -0.0334(73)
    PLMS   3  3    0.0429(34) PLMS   4  0    0.0058(44) PLMS   4  2   -0.0110(33) PLMS   4  4    0.0113(53)
```

```
ENTRY
  ID
    S405
  COMMENT
    in UBDB2018: S405 BG1
  NOI
    136
  ATOM DESCRIPTORS
#  central atom
    S1    CONNECTED_TO  O2,O3,N4,C5       PLANARITY -  PLANAR_RING_WITH_PLANAR_ATOMS -       IN_3_MEMBER_RING - IN_4_MEMBER_RING -
#  1-st neighbors
    O2    CONNECTED_TO  S1                PLANARITY *  PLANAR_RING_WITH_PLANAR_ATOMS -       IN_3_MEMBER_RING * IN_4_MEMBER_RING *
    O3    CONNECTED_TO  S1                PLANARITY *  PLANAR_RING_WITH_PLANAR_ATOMS -       IN_3_MEMBER_RING * IN_4_MEMBER_RING *
    N4    CONNECTED_TO  S1,*              PLANARITY *  PLANAR_RING_WITH_PLANAR_ATOMS *       IN_3_MEMBER_RING * IN_4_MEMBER_RING *
    C5    CONNECTED_TO  S1,*              PLANARITY *  PLANAR_RING_WITH_PLANAR_ATOMS *       IN_3_MEMBER_RING * IN_4_MEMBER_RING *
  LOCAL COORDINATE SYSTEM
    X N4 Y C5 R
  SYMMETRY
    m
  PARAMETER MODIFICATION DATE
    Fri Dec  3 10:28:39 2021
  MULTIPOLE MODEL PARAMETERS
    PVAL         5.973(41) KAPPA       0.9638(19) KPRIM       1.0437(87)
    PLMS   1 -1    0.0115(29) PLMS   1  1   -0.0084(41) PLMS   2 -2     0.021(14) PLMS   2  0     0.084(11)
    PLMS   2  2   -0.037(18) PLMS   3 -3    -0.114(26) PLMS   3 -1    -0.415(22) PLMS   3  1    -0.313(23)
    PLMS   3  3    0.284(26) PLMS   4 -4     0.085(21) PLMS   4 -2     0.193(18) PLMS   4  0     0.148(25)
    PLMS   4  2   -0.087(24) PLMS   4  4     0.166(29)

ENTRY
  ID
    P403
  COMMENT
    in UBDB2018: P403 KJ
  NOI
    27
  ATOM DESCRIPTORS
#  central atom
    P1    CONNECTED_TO  C2,O3,O4,O5       PLANARITY -  PLANAR_RING_WITH_PLANAR_ATOMS -       IN_3_MEMBER_RING - IN_4_MEMBER_RING -
#  1-st neighbors
    C2    CONNECTED_TO  P1,*              PLANARITY *  PLANAR_RING_WITH_PLANAR_ATOMS *       IN_3_MEMBER_RING * IN_4_MEMBER_RING *
    O3    CONNECTED_TO  P1,X              PLANARITY *  PLANAR_RING_WITH_PLANAR_ATOMS -       IN_3_MEMBER_RING * IN_4_MEMBER_RING *
    O4    CONNECTED_TO  P1,X              PLANARITY *  PLANAR_RING_WITH_PLANAR_ATOMS -       IN_3_MEMBER_RING * IN_4_MEMBER_RING *
    O5    CONNECTED_TO  P1                PLANARITY *  PLANAR_RING_WITH_PLANAR_ATOMS -       IN_3_MEMBER_RING * IN_4_MEMBER_RING *
  LOCAL COORDINATE SYSTEM
    X O5 Y C2 R
  SYMMETRY
    m
  PARAMETER MODIFICATION DATE
    Fri Dec  3 10:28:39 2021
  MULTIPOLE MODEL PARAMETERS
    PVAL         5.254(50) KAPPA       0.9485(33) KPRIM       1.0363(61)
    PLMS   1 -1    0.046(30) PLMS   1  1     0.232(37) PLMS   2  0    -0.168(36) PLMS   2  2     0.198(22)
    PLMS   3 -3   -0.083(17) PLMS   3 -1    -0.375(19) PLMS   3  1    -0.366(29) PLMS   3  3     0.458(32)
    PLMS   4 -4    0.071(11) PLMS   4 -2     0.259(17) PLMS   4  2    -0.058(16) PLMS   4  4     0.071(26)
```

Refcodes of the structures obtained from the CSD and used for MATTS2021 atom type parametrization.

ABABIP ABABOW ABADOX ABAFEQ ABAFUG ABAHES ABAHUI ABAJOE ABAKIZ ABALAS ABALIA ABAPAX ABAPIF ABATAB ABATOO ABEBUE ABEDOC ABEFAO ABEHEV ABEJOH ABEKAV ABEKEY ABEKEZ ABELAV ABELEZ ABEPUV ABEQUW ABEROP ABIBEU ABIDEW ABIMUT ABIPIL ABIVUD ABIVUF ABIXEO ABIXIS ABIZAM ABIZER ABOBAV ABOGOO ABOMEM ABOVEU ABUBEE ABUGOT ABUQAP ABUQOD ABUQUL ABURAQ ABUTAS ACABAK ACACOX ACADEO ACAJEV ACALIC ACAMOX ACANAC11 ACARBM01 ACAYUY ACAZOV ACBZPO01 ACECAN ACEMAX ACENAZ ACERIK01 ACETSC10 ACEVAH ACEYOX ACEYUD ACEZIR ACGLUA11 ACICUK ACIDAR ACIDOH ACIHEB ACIMDC ACINDN ACIQEI ACIQIM ACIQUY ACIRAG ACIXAN ACOREP ACRLAC02 ACSALA01 ACUKIV ACULIV ACUPUL ACUZON ACXMPR ACYHXA01 ADAGOC ADAHET ADAJUK01 ADAKUM ADALAT ADAMEY ADAVUW ADAVUX ADAWEI ADAXEJ ADAXOT ADAZOV ADEBOB ADECUI ADEKUQ ADENCH03 ADENSL ADEPAB ADESUL ADEXAJ ADHELA10 ADIHUR ADIMIK ADINAD ADIROU ADITAI ADOJAG ADOJEI ADONAK ADOPIU ADOTUJ ADPOSD ADULOA ADULUG ADUNAO ADUPOF ADUQAS ADUSEY ADUWIF ADUXAY ADUZAB AEPHOS02 AFADEQ AFAGEW AFALIC AFALOI AFALUO AFAMID AFAMOM AFAMUS AFANAZ AFANUT AFAPON AFAPUT AFAXAG AFAYOV AFAZEM01 AFAZOZ AFAZUF AFCYDP AFEBAR

AFEBEV AFEJEC AFEPIL AFETEL AFEVOW AFEWIS AFEZIV AFIFOK AFIFUR AFIGEC AFIGOM AFILEI AFINAG01 AFIPIQ AFIPOW AFIWIX AFIZAS01 AFOBIH AFOCEF AFODEF AFOTAS AFOTIA AFUGEP AFUNEW AFUQOK AFURPO10 AFUVOM AFUVUS AFUYEI AFUYIJ AFUZOT AGAGUK AGAMEA AGAMOK AGAMUQ AGASUY AGAXAJ AGEKII AGESIO AGIBAV AGIKAE AGIXOF AGOCII AGODAD AGONAN AGOXOL AGUGIU AGUGIU01 AGUPID AHADOE AHATAG AHATEK AHEBUL AHECEW AHECIB AHEJAZ08 AHETOX AHETUD AHICIF AHICUR AHIJOR AHIKEI AHOJIQ AHOWOL AHOZED AHPSUL AHPSUM AHUBEM AHUFEP AHUZEI AIPEPN20 AJACAP AJACEV AJACIX AJAFIC AJANAC AJAXAK AJEFUS AJEVAN AJEVOB AJIDAA AJIVIZ AJIWOF AJIWUL AJIXAS AJIXIC AJIXUM AJOGUC AJOPEW AJOPOE01 AJUDAL AJUNAU AKAHEZ AKALAZ AKATIQ AKAWIS AKAXAL AKEMAF AKENIO AKEQAJ AKEQOX AKETUG AKIFEF AKILAH AKIWOG AKOCAE AKOCEJ AKOCOU AKOJAN AKOPAT AKOPEX AKOQEY AKOWAY AKUMEA AKUQUT AKURAA AKUREE AKUROO AKUVEI AKUVIM ALAGUR ALANEH ALAQEK ALASAI ALAYIW ALAZAO ALECIE ALEFAZ ALEVES ALEWIZ ALOPIB ALOSIF ALOVUS01 ALUBEQ ALUGOF ALULEY ALULOJ ALUTOS AMADAU AMALUW AMANEI AMAPTZ AMAROV AMDPIM AMEFEF AMEGIK AMELIO AMELOU AMELUA AMEQOY AMIDIL AMIHXF AMIMZA10 AMITEW AMMPRA01 AMOLOF AMONIB AMONOH AMUBIV AMUBOB AMUMIG AMYGLA ANABOI ANEMEN ANISAT ANONIN11 ANOSAY ANTPYR ANTZCO ANTZOL10 ANUCUJ ANUDAQ ANUDEU ANUDOD ANURAE ANUXAJ APAPYR APAWOF APEJEM APEKEM APEKIQ APELAJ APELEN APEMOZ APIFAH APIPAS APITEA APIVIG APOLUN APORON APOXOS01 APOYIO APUFIB APUFOH APUMIJ APURAE APUZAM AQANAI AQARAN AQARIU AQEBED AQEBIH AQECAA AQEDUW AQERAQ AQEWOI AQEZUR AQIGEO AQIGOW AQINAP AQINET AQOTAC AQOZUD ARABUQ ARACYP ARADEC ARAMEN ARBIMC10 ARCLAM01 AREFIO ARFCYT10 ARGIND11 ARIFOX ARIQAV ARIXIK AROJAU AROMAV AROMID AROQEF AROVAF AROWIP ARUBIY ARUWEQ ARUYIV ASABAZ ASAXOJ ASIJIW ASIJIX ASINAS ASITAY ASIYOR ASOBOA ASOBUG ASOCOA ASOFIX ASOPAB ASORAC ASOTIM ASOTUX ASOZIT ASTROM ASULOP ASUQOW ATADUW ATAJOV ATAXUO ATAXUP ATAYEA ATDZSA04 ATESIB ATEVIF ATIBAH ATICIR ATISIH ATIXIM ATIXUY ATONOO ATOVAG ATOXEM ATUBEX ATUGAZ ATUTAK ATUTEO ATZTHD10 AVAGIN AVAKAK AVEPAU AVOTUA AVOVIQ AVUGUV AVULIM AVUSAN AVUXAS AWATUO AWAWAX AWAYOM AWAYOM01 AWEBEL AWECIO AWEFEP AWEFUD AWELOE AWENIB02 AWETED AWIQEE02 AWIXEL AWIXIP AWOLIJ AWOLOP AWOQOU AWOSOU AWUBIF AWUREQ AWUSAM AWUSOA AWUYUM AXABEH AXADUZ AXAFOV AXAJUF AXALAM AXALAN AXALOB AXAMAN AXANOD AXASUP AXAVAX AXAWUS AXAXED AXEJAQ AXEMIA AXFSUR AXIMEZ AXIQAA AXOJAZ AXOJIH AYATOK AYECAJ AYEFUF AYEXOT AYIFET AYIKID AYOHEB AYOHIF AYOHOL AYOJAZ AYOJED AYOREM AYUDUU AYUHAE AZABAF AZACUA AZAFIR AZALIX AZANUM AZAPIA AZEHOD AZEJAS AZESAZ AZHPXB AZIDES AZIDIW AZIQIJ AZOMOS AZOPIP AZOQUC AZOROX AZOSUL01 AZOTIT AZOYOE AZUCAA AZUMOY AZURAC01 AZURAP AZURUI BABKIY BACQIG BACXOV BADQIG BAFGUK BAFLOJ BAFLOL BAFTAE BAGDUK BAGTUA BAGVAI BAGYAL BAGYEO BAHGAU BAHNUU BAJYIV BAKFUO BAKLEG BAKLUW BAKSEM BAKVAL BAKVEP BALGUR BALMOR BALPIN BALSOW BALTEP BALYAQ BALYEU BAMBID BAMDIC BAMFAY BANFIG BANHOO BANHOO01 BANHOO02 BANJEG BANKUW BANLEH BANNEL BANNOU BANPAK BANREQ BANTIU01 BAPZEA BAQDEE BAQHOS01 BAQSIW BARCOX BARFIJ BASPAO BATGIN BATJUC BATSUK BATVAU BATWOI BATZOO BAVBOP BAVCAF BAWGOW BAWHEO BAXTID BAXTOJ BAYFEP BAYGOZ BAYYAB BAZNEV BCBANN01 BCZNON BDIXNA BEBLAW BEBNOM BEBWEK BEBWOU BECGOF BECLAW BECRUY BECYAL BEDBAP BEDDOE BEDJAX01 BEDJIF BEFSUA BEFWIV BEGLAC BEGPEL BEGSEL BEGVOA BEHDUO BEHLAE BEJWAO BEKBAV BEKBOJ BELMOX BELTOE BELTUK BEMDAZ BEMFOO BEMFUU BEMHAC BENMEO BENTEU BEPRAP BEPYAX BEQQOF BEQWAY BEQWEC BERBOP01 BERFUZ BERHEL BERSAS BERXAY BESLOC BESMIV BESNUI BESPUL BESYEF BETCUZ BETNET BEVFEN10 BEVLIX BEWHUG BEWQAV01 BEWQOK

BEXFUF BEXGEQ BEXLIB BEXMIA BEXWEI BEYPUR BEYVEH BEZGES BEZLUP BIBTAI BIBXUG BICBIZ BICFAX BICKOP BICSUC BIDJON BIDMIK BIFNEL BIFPIP BIFTUH BIFVET BIFXUK BIGSOZ BIGUAN01 BIGXAS BIJSIX BIKPOB BIMSNO BIMTIB BIMWAW BIOTIN10 BIPTID01 BIQTON BISDIQ BISFAN BITMIC BIVHOG BIWWIO BIXBES BIXQAB BIXQEF BIZDUK BIZWIS BIZYAL01 BOBMUB BOCDOM BOCJAF BOCPAK BOCPUE BODCED BODPAL BOGQOE BOHWUR BOLYUW BOLZUX BOMQOL BONVIL BOPBEO BOPTZO BOQJEW BOQVIM01 BOQWUZ BOQYEN BORHAS BORKID BORZUE BOSXEP BOSXOZ BOTHEY BOTZOA BOVYIV BOWDAV BOWJUT BOWTUF BOWYOC BOXGAZ BOXGIE BOXHEE BOXKEE BOXNIO BOXWOA BOXZEW BOYHIG BOYPUD BOYTEO BOYYEW BOYZUL BOZDIE BOZFUU BOZKUW BOZLIM BSULFA01 BTPMET BUBMIU BUCJER BUCJIV BUCJOB BUCKAO BUCTIF BUCVOM BUDGOZ BUDHIT BUDLUK BUDMUL BUDPEW01 BUDWAZ BUDYAB BUDYEF BUDZIM BUFGIS BUFHEQ BUFJAQ BUFLIA BUFQAX BUFQEB BUFTED BUFTEE BUFXUY BUGDEM BUGKIY BUGMIZ BUGXUW BUHHEU BUHJAP BUHJEW BUHMOJ BUHTAC BUJSUV BUJTAC BUKKUP BUKYIP BULXOX BUQCAT BUQLAC BUQMUX BURFIF BURGOL BURKUU BUSJIJ BUSPEK BUSZUM BUTFUT BUWBUR BUWSAN BUWSAO BUXFEG BUYGIN BUYLAJ BXTNON BZOXZT BZTZAD CABBAL CABPYR11 CABSIH10 CABYAG CABYAH CABZIR CACHIX CACQON CACYOU CADMAW CAFSIN CAGDIY CAGGIB CAGGOH CAGGUM CAHROS CAINSP CAKRIQ CALJIK CAMDEC CAMVAQ CAMVES CAMZOG CANCAW CANDEB01 CANELL01 CANTAO CAPARO CAPJIO CAPROA CAQYIE01 CARKOX CARYIG CARZAX CASBAA CASFAE CATXOK CAVGEL CAVQUM CAWWAB CAZGUI CBOHAZ CBOHAZ01 CBOHAZ02 CEBBIV CEBHUM CEBNIG CEBZOY CECHEY CEDDIA01 CEDGUO CEDROU CEFPUA CEFSEN CEFTOW CEGBUN CEGKEG CEGKOO CEGVER CEGYAQ CEHSIR CEJSEP CEKLAH CEKLEL CEKLIP CEKLOV CEKLUB CEKMAI CEKMIQ CEKMOW CEKNUD CEKPIT CELLAI CEPCOP CERJEP CESKUG CETLET CEVYEH CEWCAI10 CEWVUW CEXMOH CEYCIS CEYNEZ CEZQII CHPYRD CHXIQL CIBHUS CIBTUD CIBVEP CIDBIB CIDHON CIDSOA CIDXIZ CIDZUK CIDZUK10 CIFNAJ CIFNUA CIFTES CIHGAC CIHHIL CIHNOY CIJZAX CIJZUR CINCIM CINLOC CINTOK CINYUW CIPLOE CIPMOE CIPRAX CIPRIF CIPVII CIPWOO CIQCUA CIQGEP CIQHOB CIQHUH CIQJES CIQYEH CIQYEH01 CIRLEW CIRRUT CIRYIL CIRYIO CITCIU CITDAN CITJEX CITNIC10 CITPEA10 CITPEB CIVSOR CIVSUX CIWMEA10 CIWVAH CIWWAG CIXWEL CIZDUK02 CIZRUY CIZTIR CIZYEP CMPHOA10 CMTAZP CMYCZA CNOXPA COBWEY COCVIZ CODPIU CODPOA CODXUR COHBOQ COHGAH COHKOZ COHNES COJMUJ COJXIK COJXOQ COJXUW COJYAD COKJER COLLEW COMGET COMGUI01 COMXAD COPHEU COPREE10 CORSUY CORWAI COSFAR COSKAX COTQUZ COTZAP COVJEF COVXEQ COVYAQ COWXUK COXJEH COXJOR COXKAD COXSOA COXZAS10 COYROZ COZKAD COZKEH COZKIL COZSOC COZSUI CPBTSX CSURCD10 CUBDEL CUBHOZ CUBKES CUBLOD CUBVOK CUBWON CUBYIK CUCHUD CUCVAY CUDFOW CUDLEV CUFFOB CUGBAK CUGBEL CUJQED CUMQEI CUNTAG CUQCEX CUQVIU CURFEA CURPAI CUYCEF02 CUYKEM01 CUYPUH CUZQEV CYSTEA CYTIAC CYTIDI11 CYTOSC DABGOC DABKOI DABQIJ DABSOR DADWEN DAFGUP DAFJEC DAFTEK DAJWOB DAKBOJ DANGEF DAPFIJ DAPJAG DAQHOS DAQYAV DARMOB DARXOM DARZIF DASHEN DASRIY DASTAS DASYEC DAWDEN DAXCAG01 DAYKAQ DAYLAR DAYWEF DAZCLA01 DAZHOD DAZHUJ DAZPEA DAZVAB10 DBEZPO02 DEBFAR DEBHEX01 DECBOE DECGIE DECKUR DEDMUX DEDMUX01 DEDMUX02 DEDNIM DEGHED DELZAW DEMYIE DEPXUR DERFAI DERRIC DESLOD DESWUT DETBIO01 DETDOV DETFOX DETFOY DETFUC DETPUO DEVFEP DEWXIP DEXGIW DEXQOM DEXREE DEXTAC DEXTIJ DEZTEH DHPMAD DIBMEH DICPUA01 DICRAI01 DIGTET DIHRAN DIHRAQ DIHSOE DIJZIH DIKWIE DINFAH01 DINNUM DIPGIS12 DIPNOI DIQCEN DIQNOJ DISJEU DITLAT DIUREA05 DIWKUQ DIYHIC DIYPIL DNBZFX01 DNPIMZ DNPMTA DOBBIH DOCYPO03 DODNUF DODNUF01 DOFDAE DOGBIK DOJTII DOKJIY DOKWUW DOKZIN DOLBUC DOMJUM DOMQUS DOQSUX01 DORYOA DOSVOY DOTTIQ DOVTAJ DOWDEY DOWJOR DOXGEF DOXGIJ DOXPOV DOXTIW DOYMAH DOYNOU DOZKOU DOZMIO DOZRIV DUBCEI DUBSID DUCMEW DUCQAW DUCQOK

DUCXAB DUHMAW DUHWAF DUKSOR DUMXIS DUQPAH DURYAS DUSLUZ DUSXOF DUVGUX DUVHUY DUXWIC DUZYAZ DUZYED DUZYIH EKOKEU ELEPIV01 ELEYID ELINOC EQONAZ ERAHAG EVENIC EVOTUE EXOFOM FAGQOV FAGSUB FAJTUI FAKJOS FAKJUY FAKKAD FALBEA FANYAV FAPKEN FAPMUE FAQMIV FAVWIJ FAWKUL FAXPOJ FAZQOP FAZRAZ FAZRED FEBKAY FECLUU FECMEF FECQAF FEDBOF FEDXES FEDXIW FEDXOC FEDXUI FEFHUW FEFMAF FEGSEP FEGVIW FEHLOV FEHRIU FEJGIK FEJJEJ FELCIK FEPNAP FESPAU FESPEY FESPOK FETFUH FETZEJ FEVQED FEWRUV FEWSEH FEZMOO FICWAQ FICYOG FIDDII FIFGOQ02 FIGYID FIKCEI FIMPEW FIPPEB FIQKAS FIRLOK FISKIC FITJEX FIWCOF FIWNAC FLYITU FOCJOZ FOGCOU FOHKOD FOJQUQ02 FOKRUS FOKWEI FOLHAP02 FOMTOT FONFIY FONMOL FOQDIZ FORHAV FORMAC01 FOSDOG FOVNIQ FOVWAO FOWGIK FOWHIL FOWLIP FOWREP FOWVIX FOWZAS FOXFEG FOXJIO FOXPIU FOYNUC FOYXIA FPRTOD10 FUBHUI FUBWIJ FUCTIG02 FUDGIX FUDLUO FUDNUQ FUFYIP FUFZAH FUGJUM01 FUJNUW FUJPAE FUJPIM FUJYUH FULPIO FUMDUP FUMJAB FUMPUY FUNNUZ FUNXAP FUPDAX FUQSAN FUSDII FUSNOY FUSROC FUTKUC FUTLEN FUWMOA FUWTOG FUXMES FUXPIZ FUYHUC FUYMES FUYREW GABHAV GACCUK GACTEM GAFNAC GAGSIR05 GAHCEY GAHTEO GAKGEE GAKPEN GAKPIR GAKZIC GALHUX GALJEL GAMGAD GAMKEK GAMMOX GARVOK GASGOZ GATCAF GATPAS GAXMEA GEBJUU GECLUX GEDLEH GEDPIP GEFBIE GEFRAL GEFTES GEFTIW GEGSIU GEHNOZ GEJBIH GEJKUC GELGIN01 GELHIO10 GELTIA GEPPIA GEQXAE GERDOW GERFEO GETBOX GEVBEQ GEVGOF GEVHAS GEVREE GEXQEF GEYPOP GEZVUC GICCOK GIJCAD GILSOM GIMKAO GIPCAL GIPKOI GIQNOL GIQYEK GIQZOV GIRKOI GISGIZ GISLIF GITVAI GIWVOA GIXYOC GIYNEK GIZMIM GLCTSM GLPHEA20 GLUTAM02 GLYCFE03 GLYCIN85 GLYCPH01 GOCZUU GOGROL GOGZUZ GOHBUC GOHCEN GOJMOJ GOJPAW GOJPUQ GOPZIU GOQBOG GORZEV GOVSUI GOVZID GOWFEG GOWJUA GOWYOG GOWYOG01 GOWZEX GOXLOU GOXLOU01 GOXXUP GOYBAA GOYLEO GOZFAE GPHXAM GUBCUE GUBJOF GUBQAV GUCWEJ GUFRAD GUGDAO GUMGAZ GUNQUB GUPSUH GUQDUT GUQFOP GUSSOE GUSXEZ GUVCAD GUVTAS GUWCOQ GUXLAO GUYNUJ GUYPIA HABNED HABTEJ HAFHIF HAHHON HAJSAN HAKNEO HAMROE HAMZIF HANLEP HAPFOW HASVII HAYTOQ HECLUZ HEGFIJ HEGKIO HEJCEE HEKQUJ HEMYON HEPDUC HEVDAO HEWBAM HEWKAW HEXZEQ HEZVEN HIDXIB HIFQES HIFWOI HIHJUD HILHER HIMXAD HINCAL HINHUK HIRYUF HISAPH01 HISCAO HISTAN HISTCM12 HIWPOT HIXGEA HIYDIC HIYJUU HOHCOX HOHGER HOJKUO HONFIA HONLON HOPJUR HOQNEH HOQNOR HOQNUX HOQZAQ HORNUZ HORTAJ HORZAQ HORZOE HORZUK HOVGAD HOWMEO HOWYUP HOYPER HUBLIC HUGGEX HUKQEJ03 HUQROC HUQRUI HUSCAZ HUSNEP HUVZUT HUXZOR HYPRCX HYPTHO IBOCOT ICERAL ICOFAJ IDEGIK IDOHAN IDUDIU IFIDUW IFIFAE IGIHAH IGUMAY IGUMEC IHIWIH IHUVIT IJEFAE IJICIN IJIZAC IJOKEZ IKAZUQ ILICUB ILIDAI ILOPUU ILOQEH ILOZIS INAYIH INEKER INIFUI INONAB IPEWIL IRISUY01 IRODUQ IROFAY IROFEC IROFIG IROHUU ISAXUX ISOGAY ISOGII ISOGOO ITEJEY ITEMUR ITESIL ITICAR ITIGAV ITINOQ ITUTEY IVOMIR IVUHIS IWIDEZ IWILAB IWOKEM IWUXII IXAXOW IXONEQ IYAYIQ IYEZAN IYITAM IYUGOA JABCEX JABMAD JABSEN JAFJAB JAFJIK JALQET JAPJUF JAPPEV JATWUW JAVYUB JAVZEM JAWQOO JAXSEH JAYNIG JEDBUQ JEDLEK JEDVET JEKXUT JEMSUP JETYUD JIBCIG JIBDIH JICWOK JIGRAS JIHSOI JIJTII JIJXIJ JINDOZ JIPJUO JIWHUT JIYREO JIYWET JODHAN JODKEU JOGFAN JOGTAC JOPXOB JOPYUI JOSGUV JOWWIB JOXDEF JOXFIO JOXRUM JOXWUR JOZBEI JOZFIQ JUCVEL JUCVIP JUDFOG JUDWEK JUDZIU JUFDAS JUKNIO JULXEV JULZEX JUMMEL JUNBEB JUPLOX JURDIL JURLIR JURZED KACTUE KADPUA KAKLUE KAPKOC KAPWAZ KARBOV KARKUL KASNOH KAYWIR01 KECFIJ KECGOO KEDXID KEHZAY KEPHUI KEPJOE KEPNUO KEQJIA KEQPUS KERKOI01 KETLAX KETSAF KETYEN KEVQOS KEYQOX KEZREM KGULAM KICJEL KIHVEC KIKKIZ KIMNID KISGAW KISQEL KITJOP KITJUV KITREK KIWVIW KIXVUL KIZLEM KOBQEX KOCHOZ KOCNOI KOFKUL KOFLOG KOJGUM KOKBER KOMHAV01 KORYAU KOSHEI KOTPEO KOTXAU KOVCAZ KOWLIT KOWMUH KUBGUM KUHNAC KULBAW KULREP KUMZOJ

KUNBAY KUNBIG KUPGIN KUPZUS KUQBEF KUSQEW KUSQIA KUTHOW KUVYAC KUXXUY KUYBAJ KUYFIV KUYGES KUZFER LABRUB LABVIT LALNIN03 LAPFIR LARGPH06 LAVHAR LAXTIO LAXYOY LCYSTN04 LIDYEC LIQWEN LOMTEM LOPXUJ LTYRGG01 LUFBOD MABZAR MADCMP MAJDOR MAJGAG MALAMS10 MAMDEK01 MAPNUN MATDUG MAVQAE MDPIZO MEDMEO MEDMEP MEMTYR10 MEPVAE01 MEQVEK MERQUW MERXUF MHPTEC MIETDZ20 MIFMEW MIGWUV MINMUU MINOSP MINYAM MIPWEN MIQNEF MIQNIJ MISTAJ MISYES MISZUM MIVQAJ MIVQEN MIZKEN MMCPUR MNIMET05 MOBCIQ MOBYIL MOGZAK MOGZEO MOKREK MOPMOT MORSEU MOSNUG MOSRUH MOSVEY MOTJOU MOTQUK MOTROF MOXGOY MOXHAL MOZQAW MOZSOM MPHTAZ11 MPROLA10 MQAZOX MSMNIT MSULIN03 MSULIN04 MSULIN10 MSULIN11 MSULIN12 MUBDIZ MUBKIE MUBVOV MUDGAV MUGKAA01 MUHCOH MUJBIE MULFIK MUNVAS MUQZIJ MURYED MURYIH MUSYIJ MUTDOW MUXNAU MUYYUA MUZNEC NAFHUY NAGGEH NAGLEN NAJQOG NAKLOA NALCYS02 NAMZAC NAPRLA02 NARGIX NASFIX NAXCAS NECDEH NECVOG NEFGAG NEFNES NELGOB NEQMAA NEQMUU NEQNAB NEQNEF NEQNIG NEQNIJ NETGIC NEZNAH NIGTAZ NIGZIO NIKPAB NIKYEO01 NINYOB NIPBEV NIQHED NITRBE01 NIYDOO01 NMACEP01 NOKNOR NOREPH01 NORMUF NORNEQ NOSXUO NOTXUS NOVZUW NOWCAD NOXGOZ NOYLIZ NOYYOS NOZXEF NUBQOT NUBTEM NUHJIK NUHKIL NULYUP NUNFIL NUPTAV NUPVUR NURCEJ NUSMOF NUTGEO NUVHAP NUVKEW NUVKIA NUXXEJ NUXZAJ OBIDAE OCAHAC OCASER OCIMUI OCIPEV ODAPIV OFOQOR OFOYEN OGEYAA OGUGIG OHIPUR OHIWOT OJUHOS OJUHUY OKESOM OKETUT OLINEE OMOMOS OMUXEB ONUPAQ OPTHIM OQODOP OQUPEW OSEJON OVAQAF OVAQEJ OVAZUI OVUYEL OWOVED OZIZEE PABSOC PAEXPY PAEXPY01 PAEXPY02 PAGXOL PAKWON PAQPEE PAQPII PAWDAV PECHEN01 PECXIG PEFDOT PEGJIV PEKZEK PELQAY PELWOS PEQCEV PEQCIZ PEQCOF PEQDAS PEQDEW PEQDIA PEWHEE PEXTAN PEYSES PEZPIU PEZPUG PHOGLY04 PIBGIS PILMAZ PINPAD01 PIPGON PIYKIR PIYMUI PIZXAA POBMIC POBSAB POJQEK POMDAW POMDAW01 PONTIV POTHRE01 POVKIU POXVAC POYJUL PROAPH02 PUGBEZ PUHKOV PUJBUU PUJNUG PUJXUQ PUKVOI PUMYIH PUNJOZ PURBAH PURBEL PURQOK PURREB PURROM PUSVIK PUSYOT PUXKAU PUZZAL PUZZEP PYRIDS02 QACLAH QADRIY QAFTUN QAKWAB QANJUL QAXDEY QAXDIC QAXLIK QAXMEH QEBBUW QEBYUR QECCIK QECNIV QEKXUZ QEPLUV QESRIP QEVPEM QEZLOW QIJDAO QIJDAO01 QIJDAO02 QIJHUM QIMPEK QINGAV QIPBOJ QIPKEI QIQHIK QOHDIC QOKWAP QOTNAP QOVXEF QOYJOD QOYJOD01 QOYJOD02 QOYJOD03 QOYJOD04 QOYJOD05 QOYJOD06 QOYJOD09 QOYXIO QOZTIK QUBLIL QUBVUE QUBWUI QULHEM QULVEY QUNZAC RADGAF RADKOX RADZOL RAFBAB RAJFIR RAJXUW RAMVOQ01 RAWFOM RAXKAD RDCPLB01 RECZIJ REDTUR REFCIP REMDOF RENNOQ01 RENPOS REQQUA RESTOY REVCEB RICFUG RIPGEE RIQKEH RISCUR RIWBUV RIYDOV RIYYAA RODFAR ROKQUD ROWZOV ROZPUT RUBBAU RUFQUH RUGZUR RUHBEE RUKCEH RULHIR RUNGAK RUSCOZ RUWFEU SADMEQ SADMUG SAFFEK SAHBUZ SAKHUI SANPEE SAPTUZ SARZAM SASBIX SATCEW SATNAC SATNEH SAVSOX SAYWAQ SAZCOL SCCHRN06 SCGMPT10 SEDBUZ SEDZOQ SEDZOQ01 SEDZOQ02 SEGGER SEJSIJ SEKWIO SERPOP01 SETCOK SEWGEG SEZYIH SIHPAB SIJQEK SIRGUY SIRRET SIRRIX SIRROD SIXWUS SIYLOB SIZTON SIZXAA SLFSMD11 SOFMEG SOJDEA SOLVIZ SONDII SOPLUH SOPMAO SOYPUT SUFNUC SUFYOK SUGFIJ SUGGUZ SUHSOG SULBOS SULSIC SUMPAR SUNWUV SURREC SURTAA SUXDOE TAHJIV TAURIN11 TAWSIU TEDZUY TEFZUB TEJDAN TEJMOK TEJNEB TEKNAY TEQZAR01 TERMIM TERNOT TERYAQ01 TERYAQ02 TEVHEH01 TEVWIB TFMSAD TFSLAC TIGWIQ TIGWIQ01 TIRPAL TIXPIZ TMEYPH TODNUV TOHWIW TONQAO TOPBIK TOSWUW TOVBUE TOWKAU TOXTOS TOXTUY TOZLOJ TPHCUR TPYPOP10 TUBERC01 TUDMOU TUDZEY TUDZIC TUGWUN TUGXAU TUNTUT TURYIQ TURZEN TUSVEK TUWVOY TUYWUH UHAWUV UNOCAB VACQIC VAFSOM VAFVUU VALTUX VANREH VANRIL VAPZOB10 VARNIM VASSAL VEFZIP VEGKIB VELTIQ VENHUS VIGNII VIJKUX VIKHON VINXAS VIOLME VIRYEA VISDIK VISDUZ VISVIC VIVPEW VOCKIH VOJJIN VOMKOZ

VOMZAY VOSLEX VOVMIF VOVSAA VOWGAP VOWNAZ VOWTEG VOYPOR VOZHAW VUBVUM VUCMEO VUCRIX VUKQUO VUKRAV VUMMIZ VUMMOF VUMMUL VUPJUM VUWCEW VUXKAA VUXKEE VUXKII VUXKOO VUXLAB VUXLEF VUYMAD WAFROK10 WAKWUB WALFOG WALWOW WAZDEG WEBFIS WEJBIW WEKHEA02 WELWUF WEPMAH WEPXAT WEPXEX WETNAK WEVKEO WIFREI WILJIN WINFEH WINPUH WIPCAZ WISGAJ WIZZEL WOBTUE WOBXAM WOCSAI WOGWEV WOKPAO WOLRUK WOMCAC WOMYEC WONJAK WOPJIU WOQZOR WOTPON WOTZEK WOVXOU01 WOWNOO WOZMUW WUBTOF WUCZOL WUKWEF WUKWIJ WUQPAB WUSJEZ WUXBIA XACQEZ XAFPUR XAFQAY XAFQEC XAJNIG XAKZIS XAKZOY XANOPT XANZUJ XATXEV XAZWOL XEGLAW XEKRIR XIFRUA XIHSEM XIRVAY XODXOF XOJJUD XOPNEV XORNOH XORXOU XOSJAT XOSQAA XOSRUV XOTNAY XOTRIK XOVHAU XOWCAQ XOWGOF XOYFIA XOYLEF XOYSEM XOYSIQ XOZPUA XUCHAE XUCHUB XUCQAN XUHQUM XUHXEG XUJKUL XUJWUU XULZUC XUQPIK XURNIJ XUSTUC XUTCEW XUTDEX XUVMUX XUZJUX01 YAJZUF YAKQOS YAKREJ YALLON YANQEK YAWNIT YAYDAD YAYSOH YEHYOZ YEMFOL YENKEH YEQCIG YIHHAY YIJMUC YIMPIU YIRHUF YIRNOC YISKIU YIZCOZ YIZCOZ01 YOKLUI YOMZEI YOPSUR YOPZOT YOSYEN YOTREH YOZZOF YUBNIV YUDBAC YUDBEG YUDJOZ YURNAA ZAQWIZ ZAYJOY ZAYPAQ ZEDJEX ZEHKEC ZEJMOQ ZETKUE ZIBLII ZIDYET ZIKFOO ZILHOR02 ZIVKOE01 ZIZJOH ZIZKAU ZONFOX ZOVPEI ZOVQAF ZOVQEJ ZOZWAM ZUBDAD ZUBXOM ZUGPUM ZUHBUC ZUHCIR ZUHHIT ZUHHOZ ZUHJAN ZUJFIW ZUJRII ZUJSEF ZUJWEJ ZUJWIN ZUMHUM ZUPPAD ZUPRIN ZURLOP ZUSXAO ZUTFOL ZUTKAC ZUTKEG ZZZFKK03 ZZZSBA ZZZSPS ZZZUEE04

References

(1) Cordero, B.; Gómez, V.; Platero-Prats, A. E.; Revés, M.; Echeverría, J.; Cremades, E.; Barragán, F.; Alvarez, S. Covalent Radii Revisited. *J. Chem. Soc. Dalt. Trans.* **2008**, No. 21, 2832–2838. https://doi.org/10.1039/b801115j.

(2) Haneef, I.; Moss, D. S.; Stanford, M. J.; Borkakoti, N. Restrained Structure-factor Least-squares Refinement of Protein Structures Using a Vector Processing Computer. *Acta Crystallogr. Sect. A* **1985**, *41* (5), 426–433. https://doi.org/10.1107/S0108767385000915.

(3) Urzhumtsev, A. G. How to Calculate Planarity Restraints. *Acta Crystallogr. Sect. A* **1991**, *47* (6), 723–727. https://doi.org/10.1107/S0108767391006268.

(4) Volkov, A.; Li, X.; Koritsanszky, T.; Coppens, P. Ab Initio Quality Electrostatic Atomic and Molecular Properties Including Intermolecular Energies from a Transferable Theoretical Pseudoatom Databank. *J. Phys. Chem. A* **2004**, *108* (19), 4283–4300.

(5) Dominiak, P. M.; Volkov, A.; Li, X.; Messerschmidt, M.; Coppens, P. A Theoretical Databank of Transferable Aspherical Atoms and Its Application to Electrostatic Interaction Energy Calculations of Macromolecules. *J. Chem. Theory Comput.* **2007**, *3* (1), 232–247.

(6) Jarzembska, K. N.; Dominiak, P. M. New Version of the Theoretical Databank of Transferable Aspherical Pseudoatoms, UBDB2011--towards Nucleic Acid Modelling. *Acta Crystallogr. Sect. A Found. Crystallogr.* **2012**, *68* (1), 139–147.

(7) Volkov, A.; Macchi, P.; Farrugia, L. J.; Gatti, C.; Mallinson, P.; Richter, T.; Koritsanszky, T. XD2006--A Computer Program for Multipole Refinement. *Topol. Anal. Charg. Densities Eval. Intermol. Energies from Exp. or Theor. Struct. Factors* **2006**.
